# Active predation, phylogenetic diversity, and global prevalence of myxobacteria in wastewater treatment plants

**DOI:** 10.1038/s41396-023-01378-0

**Published:** 2023-02-11

**Authors:** Lu Zhang, Xinyu Huang, Jizhong Zhou, Feng Ju

**Affiliations:** 1 Research Center for Industries of the Future, Westlake University, Hangzhou, Zhejiang China; 2 Key Laboratory of Coastal Environment and Resources of Zhejiang Province, School of Engineering, Westlake University, Hangzhou, Zhejiang China; 3 Institute of Advanced Technology, Westlake Institute for Advanced Study, Hangzhou, Zhejiang China; 4 Westlake Laboratory of Life Sciences and Biomedicine, Hangzhou, Zhejiang China; 5 Institute for Environmental Genomics, Department of Microbiology and Plant Biology, University of Oklahoma, Norman, OK USA

**Keywords:** Microbial ecology, Biodiversity, Environmental microbiology, Microbial ecology, Environmental sciences

## Abstract

The operation of modern wastewater treatment plants (WWTPs) is driven by activated sludge microbiota, a complex assemblage of trophically interacting microorganisms. Microbial predation is crucial to fundamental understanding of how biological interactions drive microbiome structuring and functioning of WWTPs. However, predatory bacteria have received little attention regarding their diversity, activity, and ecological function in activated sludge, limiting the exploitation of food web interactions for wastewater microbiome engineering. Here, by using rRNA-stable isotope probing of activated sludge microbiota with ^13^C-labeled prey bacteria, we uncovered diverse as-yet-uncultivated putative predatory bacteria that actively incorporated ^13^C-biomass. Myxobacteria, especially *Haliangium* and the mle1-27 clade, were found as the dominant active predators, refreshing conventional views based on a few predatory isolates of *Bdellovibrionota* from WWTPs. The identified predatory bacteria showed more selective predation on prey compared with the protists dominated by ciliates, providing in situ evidence for inter-domain predation behavior divergence in activated sludge. Putative predatory bacteria were tracked over a two-year microbiome monitoring effort at a local WWTP, revealing the predominance of *Myxococcota* (6.5 ± 1.3%) over *Bdellovibrionota* (1.0 ± 0.2%) lineages. Phylogenetic analysis unveiled highly diverse myxobacteria inhabiting activated sludge and suggested a habitat filtering effect in global WWTPs. Further mining of a global activated sludge microbiome dataset revealed the prevalence of *Myxococcota* (5.4 ± 0.1%) species and potential impacts of myxobacterial predation on process performance. Collectively, our findings provided unique insights into the predating activity, diversity, and prevalence of *Myxococcota* species in activated sludge, highlighting their links with wastewater treatment processes via trophic regulation of enteric and functional bacteria.

## Introduction

Microorganisms play a central role in mediating the wastewater decontamination of a large variety of organic carbon and inorganic nutrients (i.e., nitrogen and phosphorus) in wastewater treatment plants (WWTPs) [1, 2]. Activated sludge comprises diverse organisms, including bacteria, fungi, protists, and bacteriophages, which interact intimately with each other underlying the sludge function [3, 4]. Predation is a key component of microbial interactions and could exert strong impacts on biological process functioning in WWTPs, such as carbon mineralization and pollutant removal in activated sludge [5, 6]. Predatory bacteria could prey on other bacteria, similar to protists and bacteriophages but with distinct predating strategies [7]. However, intra-bacterial predation has been largely neglected in prior studies on wastewater treatment microbiota, although predatory bacteria are ubiquitous in environment and particularly regarded as “living antibiotics” due to their capability to kill a range of human and plant pathogens [8–10]. Compared with the well-studied protozoa [11, 12] and bacteriophages [13, 14], predatory bacteria, especially their grazing behaviors on the beneficial (e.g., carbon and nitrogen-removing) or detrimental (e.g., antibiotic resistant and pathogenic) bacteria in WWTPs, remain largely unexplored, which impedes a comprehensive understanding of food web interactions therein and development of specific engineering solutions, based on such intricate ecological interactions, to prevent system performance deterioration, solve long-standing operational problems (e.g., bulking and foaming), or tackle sanitation challenges (e.g., COVID-19 and antimicrobial resistance).

Predatory bacteria hunt actively and kill their prey bacteria to obtain nutrients and energy, with different hunting strategies and prey ranges [8, 15–17]. Intra-bacterial predation has been proposed as an important selection force in different ecosystems which shapes the bacterial community composition [18–20]. The best-known groups of predatory bacteria are affiliated with *Bdellovibrionota* and *Myxococcota* [21], both of which formerly belonged to *Proteobacteria* but were recently reclassified as phylum-level lineages according to a genome-based taxonomy [22]. *Bdellovibrionota* species dwell in both aquatic and terrestrial environments and are known as obligate predators [23], whereas soil is the major habitat for members of *Myxococcota* which are mostly facultative predators [24, 25]. From WWTPs, a few prior studies isolated *Bdellovibrio*-and-like organisms (BALOs) and found they could alter microbiota composition of both activated sludge flocs and granules [26] and possibly facilitate waste sludge biolysis [27]. However, *Myxococcota* species were rarely functionally characterized in activated sludge, although they became frequently reported in recent metagenomic surveys [4, 28–30]. For example, a *Haliangium*-associated myxobacterium was recently recognized as one of 28 core bacterial lineages in activated sludge of 269 global WWTPs [2]. Furthermore, meta-analysis of myxobacterial diversity revealed that activated sludge accounted for 6.80% (only secondary to 59.42% by soil) of the current publicly available myxobacterial 16S rRNA gene sequences, and thus could be a promising but yet underexplored reservoir for obtaining novel myxobacterial strains [31]. Contrastingly, little is known about the activity, diversity, and functions of myxobacteria in activated sludge.

The existing knowledge about diversity, predation lifestyles, and environmental consequences of predatory bacteria in diverse environments has been mostly derived from targeted cultivation and predation experiments with isolates, which excludes access to the as-yet-uncultivated diversity of bacterial predators in environment. Stable isotope probing (SIP), since its first application [32], has been widely used for identifying microorganisms in complex environments involved in diverse metabolic pathways and biogeochemical cycling processes in situ [33–36]. This technique identifies microorganisms that consume a specific substrate labeled with a stable isotope (e.g., ^13^C, ^15^N, and ^18^O) by physically separating the labeled nucleic acids with shifted buoyant density. In recent years, DNA- and RNA-SIP has shown its potential in identifying key microbial food web players and elucidating trophic interaction patterns without the need for microbial cultivation [19, 37–41]. Especially, RNA-SIP, independent of cellular replication, is more sensitive in detecting microbial activities involved in targeted metabolic and ecological processes [42]. Thus, RNA-SIP through incubation with isotopically labeled prey cells could serve as a powerful approach to advance our understanding of microbial predation and ecology in natural and engineered environments, such as activated sludge where whether myxobacteria are active food web constituents and how trophic interactions could affect microbiota performance in biological wastewater treatment and resource cycling awaits exploration.

In this study, we aimed to explore the diversity of active predators on typical detrimental and beneficial bacteria in activated sludge by employing rRNA-SIP. We argued that predatory bacteria (e.g., members of *Bdellovibrionota* and *Myxococcota*) should compose an active part of the ecological network of microbiota interactions in activated sludge and hypothesized that they could show prey preference compared to protists, commonly regarded as generalist predators, in such complex engineered systems. Driven by our intriguing findings in activated sludge microcosms that members of *Myxococcota* dominated the potential bacterial predators consuming ^13^C-labeled prey biomass, we tracked the distribution of *Myxococcota* and *Bdellovibrionota* genera in situ in a local WWTP, projected their worldwide prevalence in activated sludge microbiota using a published microbiota dataset by the Global Water Microbiome Consortium [2], and explored their potential influence on activated sludge functioning, which jointly suggested the predominant roles of myxobacteria in microbial predation in activated sludge. The comprehensive microcosm-to-field insights obtained in this study revealed the previously overlooked intra-bacterial predation in activated sludge and highlighted the ecological and engineering relevance of myxobacteria in wastewater treatment systems.

## Materials and methods

### Sludge sampling

Sludge samples for microbiome monitoring were obtained from the aeration and anaerobic tanks at a local municipal WWTP in Hangzhou (WWTP01), China, from March 2019 to April 2021 (eight samplings, Supplementary Table S1) at the same sampling points. The activated sludge used in the microcosm experiment was sampled from an aeration tank and transported to the laboratory in a cooling box (~4 °C) within one hour, before the sludge microcosms were set up immediately. More details are available in Supplementary Method S1.

### ^13^C-labeling of prey bacteria and microcosm incubation

Two bacterial strains, i.e., *Escherichia coli* ESS5, as representative enteric bacterium, and *Pseudomonas putida* ESE1, as indigenous nitrogen-removing bacterium, both isolated from the sampled WWTP [43], were used as prey bacteria in the SIP microcosm experiment. These two strains were grown in minimal salt medium (MSM; Coolaber) containing 4 g l^−1^ 99% ^13^C_6_-glucose (Cambridge Isotope Laboratories, Inc.) as the sole carbon source. The same strains were cultivated in MSM medium with ^12^C-glucose (Sigma Aldrich). Bacterial cells were transferred once with the same medium, harvested, washed, and resuspended in MSM. ^13^C-labeling of the harvested bacterial cells was determined by a gas isotope ratio mass spectrometer (IRMS; MAT 271, Thermo Scientific) at Shanghai Research Institute of Chemical Industry.

The ^13^C-labeled *E. coli* and *P. putida* cells were added to 10 ml of activated sludge to achieve a targeted concentration of 2 × 10^8^ cells ml^−1^ in glass bottles closed with screw cap and rubber stopper. In parallel, ^12^C-*E. coli* and *P. putida* cells were added to the ^12^C-microcosms and a control group without cell amendment was set up. Bottles were destructively sampled with duplicate microcosms after 16 h, one day, two days, four days, and eight days of incubation, resulting in a total of 50 glass bottles. The microcosms were incubated at room temperature with shaking at 120 rpm. Sampled sludge was immediately frozen in liquid nitrogen and stored at −80 °C. More details on ^13^C-labeling of prey bacteria and microcosm incubation were provided in Supplementary Method S2 and S3. ^13^CO_2_ production stemming from ^13^C-labeled bacterial biomass in microcosms was monitored via daily measurements with GC-MS (Trace1300-ISQ7000 GC-MS, ThermoFisher), as described in Supplementary Method S4.

### RNA extraction, gradient centrifugation, and RT-qPCR quantification

Total RNA and genomic DNA were simultaneously extracted from all the microcosms using RNeasy PowerSoil Total RNA Kit and RNeasy PowerSoil DNA Elution Kit (Qiagen). The extracted RNA or DNA from the duplicate microcosms was pooled for each treatment at each sampling time point for the subsequent SIP and sequencing analyses. SIP analysis was performed for samples collected after 16 h, one day, two days, and four days of incubation, following the protocol described by Lueders [44]. Gradient mixture of cesium trifluoroacetate (CsTFA; GE Healthcare), Hidi formamide (Applied Biosystems), and gradient buffer containing 1 μg of purified RNA in 5.1 ml seal tubes was centrifuged on a P65VT2 rotor in a CP100NX ultracentrifuge (all Hitachi) at 125 000 g_av_ for ~64 h. Thirteen fractions were collected from each gradient according to the procedures described by Lueders [44]. RNA was retrieved from all the fractions by precipitation with isopropanol, washed with 70% ethanol, and resuspended in TE buffer. More details on RNA extraction and gradient centrifugation are available in Supplementary Method S5. The quantitative distribution of the retrieved rRNA gene transcripts across the fractions of each gradient was determined with quantitative reverse transcription PCR (RT-qPCR) of 16S rRNA gene transcripts as described in Supplementary Method S6.

### Sequencing and isotopically labeled microbe identification

Based on the quantitation results of 16S rRNA gene transcripts across gradients, a representative “heavy” (density 1.851–1.872 g ml^−1^) and a “light” (density 1.805–1.819 g ml^−1^) fraction were selected from each gradient (Supplementary Fig. S1) for the subsequent sequencing analysis to evaluate the enrichment of microorganisms in ^13^C-heavy fractions. The complementary DNA (cDNA) was synthesized, and bacterial 16S rRNA gene V3-V4 region and eukaryotic 18S rRNA gene V4 region were amplified. Amplicon libraries were constructed and sequenced on the NovaSeq platform (Illumina) at the Guangdong MagiGene Biotechnology Co., Ltd. (Guangzhou, China). The rRNA gene amplicon sequence data were processed using the QIIME 2 pipeline [45] and DADA2 algorithm [46]. Taxonomy was assigned to amplicon sequence variants (ASVs) using the SILVA SSU database (version 138) [47]. For details on the gradient fraction sequencing and sequence analysis, consult Supplementary Method S7. The final ASV tables for the 16S and 18S rRNA gene datasets comprised 16 671 bacterial and 5 258 eukaryotic ASVs, respectively.

To identify the ASVs and microbial genera that incorporated the ^13^C-labeled *E. coli* or *P. putida* biomass, we employed an “enrichment factor” (EF) to infer the enrichment of the corresponding sequences in ^13^C-heavy fractions as previously applied [40, 48] and calculated as follows:

1 $${{{{{{{\mathrm{EF}}}}}}}} = \frac{{13{{{{{{{\mathrm{c}}}}}}}}_{{{{{{{{\mathrm{Heavy}}}}}}}}}}}{{13{{{{{{{\mathrm{c}}}}}}}}_{{{{{{{{\mathrm{Light}}}}}}}}}}} - \frac{{12{{{{{{{\mathrm{c}}}}}}}}_{{{{{{{{\mathrm{Heavy}}}}}}}}}}}{{12{{{{{{{\mathrm{c}}}}}}}}_{{{{{{{{\mathrm{Light}}}}}}}}}}}$$

where Heavy and Light are the taxon-specific relative sequence abundance in the selected heavy and light fractions of a gradient, and ^13^C and ^12^C in this formula refer to the treatments with ^13^C-labeled and unlabeled cells. EFs were calculated for all the prokaryotic and eukaryotic genera with >1% read abundance, and ASVs with >0.1% read abundance in heavy rRNA fractions of at least one treatment at a sampling time point. Microbial genus-level clades and ASVs with an EF > 0.1 were regarded ^13^C-labeled in this study. As no usable sequencing data was produced from the heavy fractions of the ^12^C-gradients of the *E. coli*-16 h as well as *P. putida*-16 h and 2 d groups potentially due to too low RNA quantities, we presented results only for the *E. coli*-microcosms after one day, two days, and four days of incubation, and *P. putida*-microcosms after 1 d and 4 d.

### DNA extraction, PCR amplification, and sequencing

To reveal the in situ abundance of the putative predatory bacteria identified in the SIP microcosm experiment, genomic DNA was extracted from the aerobic and anaerobic sludge samples collected from WWTP01 and sequenced as described in Supplementary Method S8. Briefly, bacterial 16S rRNA gene V3-V4 regions were amplified and sequenced on the NovaSeq platform. Full-length bacterial 16S rRNA genes were amplified and sequenced on Sequel II platform (PacBio). Amplicon library construction and sequencing were performed at the Guangdong MagiGene Biotechnology Co., Ltd. (Guangzhou, China).

### Phylogenetic analyses of full-length bacterial 16S rRNA gene sequences

To characterize the diversity of *Myxococcota* and *Bdellovibrionota* species in WWTPs, full-length 16S rRNA gene sequences of *Myxococcota* and *Bdellovibrionota* species obtained in this study were combined with sequences acquired from the MiDAS database, a global catalog of WWTP bacterial 16S rRNA gene sequences [30], and subjected to phylogenetic analysis. Cross-habitat diversity of selected myxobacterial genera was further explored by applying phylogenetic analysis to the 16S rRNA gene sequences of WWTP01 together with sequences from the SILVA database [47] with known isolation source. Details on the phylogenetic analysis are available in Supplementary Method S9.

### Global activated sludge microbiome dataset and analyses

To explore the distribution of taxa related to *Myxococcota* and *Bdellovibrionota* in activated sludge on a global scale, a microbiome dataset of global WWTPs was acquired from the Global Water Microbiome Consortium [2]. We applied taxonomy assignment to representative sequences of the 96 148 OTUs (97% sequence identity cutoff) obtained in this study using the SILVA SSU database (version 138) [47] in QIIME 2 [45]. Specifically, abundance data of *Myxococcota* and *Bdellovibrionota* genera, were extracted and investigated for the correlation to sludge performance, abundance of functional bacteria, and sludge process parameters. More details on the global activated sludge microbiome dataset and data analysis performed in the current study is available in Supplementary Method S10.

## Results

### Myxococcota and Bdellovibrionota were active constituents of activated sludge microbiota

**Fig. 1:**
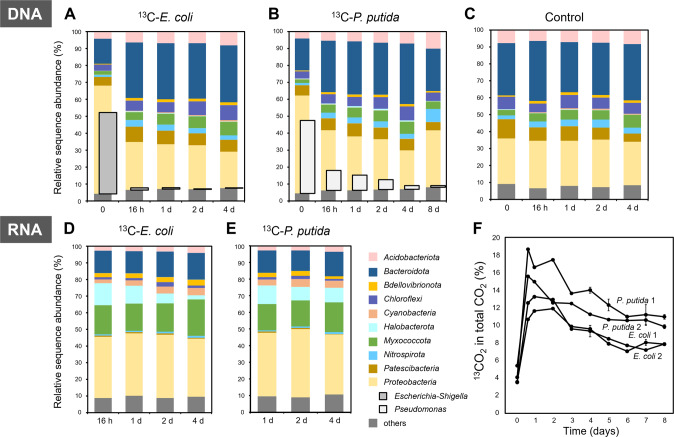
The dynamics of the prokaryotic communities and mineralization of the added ^13^C-biomass during the microcosm experiment. The overall prokaryotic communities were obtained by 16S rRNA gene amplicon sequencing of the total DNA from the activated sludge microcosms amended with ^13^C-*E. coli* (**A**) and ^13^C-*P. putida* (**B**) cells, and the control group (**C**) without amendment. The structure of the active prokaryotic communities was inferred based on amplicon sequencing of the light rRNA fractions from the microcosms amended with ^13^C-*E. coli* (**D**) and ^13^C-*P. putida* (**E**) cells. The temporal change in the proportion of produced ^13^CO_2_ in total CO_2_ indicated the mineralization of the ^13^C-labeled cells of *E. coli* and *P. putida* in duplicate microcosms (**F**). Relative sequence abundance of the ten most abundant prokaryotic phyla, together with the genera *Escherichia-Shigella* and *Pseudomonas*, was shown.

To explore the predating activity and diversity of predatory bacteria in activated sludge, ^13^C-labeled *Escherichia coli* and *Pseudomonas putida* cells (determined as 97.09 and 97.30 atom% ^13^C, respectively) were added to the sludge microcosms for maximumly eight days of incubation, and ^13^C incorporation was examined using rRNA-SIP to identify prokaryotic and eukaryotic microorganisms involved in actively consuming the ^13^C-labeled prey cells. Bacterial 16S rRNA gene amplicon sequencing-based analysis indicated the relative contribution of 47.9% and 42.7% of the obtained sequences by the added biomass upon amendment in the ^13^C-*E. coli* (Fig. 1A) and ^13^C-*P. putida* (Fig. 1B) microcosms, which dropped below 1.0% after 16 h and eight days of incubation, respectively. The overall bacterial community structure at the steady state was highly comparable to that of the control microcosms (Fig. 1C), indicating that the prey cell amendments did not induce too strong fluctuation in the microbiota structure during the SIP experiment that prevented disentangling the indigenous community dynamics.

The metabolically active bacterial communities, as inferred by 16S rRNA gene transcripts of the light rRNA fractions from the microcosms, were rather consistent throughout the experiment (Fig. 1D, E), but they showed clear compositional differences compared to the overall prokaryotic communities inferred by 16S rRNA gene amplicon sequences (Fig. 1A, B). *Myxococcota* and *Bdellovibrionota* species showed an average relative abundance of 17.5 (±0.7) % and 2.7 (±0.2) % in the 16S rRNA gene transcripts, respectively, which were significantly higher than 5.4 (±0.6) % and 1.3 (±0.1) % in the 16S rRNA genes of bacterial communities (*p* < 0.001, Mann–Whitney *U* test), suggesting their relatively high metabolic activity in the microcosms. The same activity pattern was observed for *Cyanobacteria* (3.8% vs. 0.4%), *Proteobacteria* (37.4% vs. 28.4%), and archaeal *Halobacterota* (8.6% vs. 0.2%). In contrast, members of *Acidobacteriota*, *Bacteroidota*, *Chloroflexi*, *Nitrospirota*, and *Patescibacteria* were much less abundant (average rRNA /rRNA gene ratio: 0.10–0.49) in the active communities compared to in the overall microbiota. The relative proportion of ^13^CO_2_ derived from the ^13^C-labeled biomass peaked already in 16 h or 1 d after cell amendment, indicating the rapid flow of the added biomass ^13^C into the microbial food web (Fig. 1F).

For the micro-eukaryotic communities, the added prey bacterial cells, as expected and afore-demonstrated for bacterial communities, did not exert large change in community structure inferred from 18S rRNA gene amplicon sequences, whether for ^13^C-*E. coli* or ^13^C-*P. putida* (Supplementary Fig. S2). Several micro-eukaryotic groups were metabolically active members in the whole community. For example, *Ciliophora* contributed to 55.2–78.4% of the 18S rRNA gene transcripts in all the microcosms (Supplementary Figs. S2D, E), while they were much less abundant (6.5–37.0% of the 18S rRNA gene sequences) in the whole micro-eukaryotic communities (Supplementary Figs. S2A, B). By contrast, fungal *Cryptomycota* accounted for 37.3–60.6% in the micro-eukaryotic communities (Supplementary Fig. S2A, B), while they constituted only 1.7–4.6% of the 18S rRNA gene transcripts (Supplementary Figs. S2D, E).

### Myxococcota dominated ^13^C-biomass incorporators in activated sludge

**Fig. 2:**
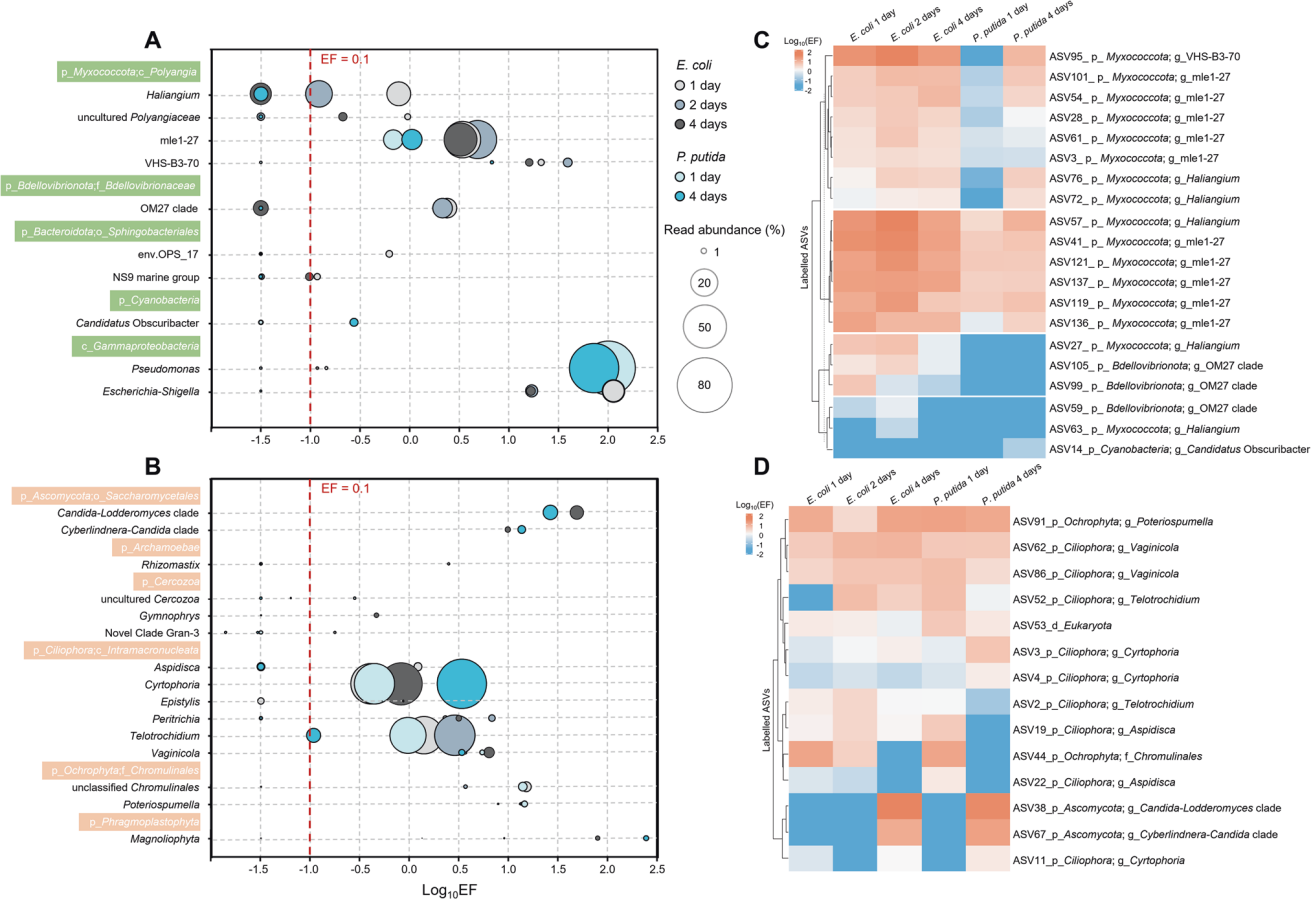
The enrichment of incorporators of added ^13^C-biomass in heavy rRNA fractions and the temporal labeling patterns. ^13^C-labeled prokaryotic (**A**) and micro-eukaryotic (**B**) genus-level taxa were identified by SIP in the microcosms added with *E. coli* and *P. putida* after one, two, and four days of incubation. Enrichment factor (EF) was calculated for microorganisms using heavy and light rRNA gradient fractions of the ^13^C- and ^12^C-microcosms to infer ^13^C-labeling. Taxa with an EF > 0.1 in at least one of the treatment groups at one sampling time point was considered labeled. The area of circles indicates the relative sequence abundance of the labeled taxa in heavy ^13^C-rRNA. The negative EFs and positive EFs < 10^−1.5^ were arbitrarily set to 10^−1.5^ for a neat graphical display. Heatmap of the ^13^C-incorporating prokaryotic (**C**) and micro-eukaryotic ASVs (**D**) with relative sequence abundance >1% in the heavy rRNA fractions of at least one of the ^13^C-*E. coli* and ^13^C-*P. putida* microcosms at a sampling point.

To predict predator-prey interactions in activated sludge, the degree of ^13^C-labeling of each prokaryotic or micro-eukaryotic taxon was quantitively determined relative to the ^12^C-control group by calculating the taxon-specific enrichment factor (EF) (see Method for calculation). Most of the ^13^C-labeled prokaryotic ASVs (as a proxy for species) belonged to *Myxococcota*, followed by *Bdellovibrionota* (Supplementary Fig. S3A). Among the bacterial genera with relative abundance >1% in the ^13^C-heavy fractions, strong ^13^C-labeling was found for the as-yet-uncultivated myxobacterial mle1-27 clade (average EF 2.66 across time and treatments), which contributed to 10.3% to 38.9% of the 16S rRNA gene transcripts in the ^13^C-heavy fractions, indicating its high metabolic activity in consuming the ^13^C-labeled biomass of both *E. coli* and *P. putida*. Comparatively, *Haliangium* spp. and uncultured *Polyangiaceae* belonging to *Myxococcota*, as well as the as-yet-uncultivated OM27 clade belonging to *Bdellovibrionota*, also exhibited strong ^13^C-labeling (maximum EF across time: 2.4–39.5), but almost exclusively in the microcosms amended with ^13^C-*E. coli* cells (Fig. 2A). The as-yet-uncultivated myxobacterial VHS-B3-70 clade exhibited the strongest enrichment (average EF 16.67 across time and treatments) but made up only 0.2% to 2.3% of 16S rRNA gene transcripts of the ^13^C-heavy fraction (Fig. 2A). Overall, our microcosm experiment tracking added ^13^C-labeled prey bacterial cells with rRNA-SIP suggested prominent predatory activity of *Myxococcota* and *Bdellovibrionota* lineages including largely as-yet-uncultivated ones (e.g., the mle1-27, VHS-B3-70, and OM27 clades) in activated sludge.

### Myxococcota and Bdellovibrionota predated more selectively than protists

**Fig. 3:**
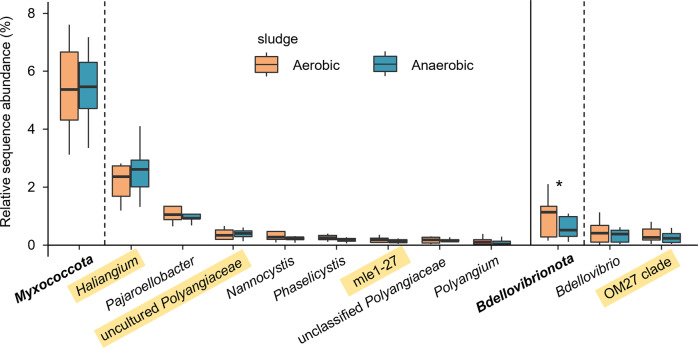
In situ relative abundance of *Myxococcota* and *Bdellovibrionota* in aerobic and anaerobic sludge at a local WWTP (WWTP01) based on sampling over two years. The abundance of the abundant genera belonging to *Myxococcota* and *Bdellovibrionota* in aerobic and anaerobic sludge were compared according to amplicon sequencing-based analysis of bacterial 16S rRNA gene V3-V4 region. The top 10 abundant genus-level taxa across samples collected from eight samplings are shown, with the putative predators identified by SIP in the microcosm experiment highlighted. The asterisk denotes significant difference in relative abundance between aerobic and anaerobic sludges (*p* < 0.05, paired samples Wilcoxon test, *n* = 8).

For the micro-eukaryotes, several taxa belonging to *Ciliophora*, especially *Cyrtophoria* spp. and *Telotrochidium* spp., and also *Peritrichia* spp., *Vaginicola* spp., *Aspidisca* spp., and *Epistylis* spp., were highly enriched (maximum EF across time and treatments: 0.9–6.7) in the ^13^C-heavy rRNA fractions (Fig. 3B), in agreement with the dominance of *Ciliophora* in the micro-eukaryotic rRNA gene transcripts (Fig. 2B). The *Candida*-*Lodderomyces* clade and *Cyberlindnera*-*Candida* clade within *Ascomycota*, *Magnoliophyta* spp. within *Phragmoplastophyta*, and *Poteriospumella* spp. and unclassified *Chromulinales* within *Ochrophyta* were also strongly labeled (maximum EF: 13.5–242.5, Fig. 2B). Moreover, the ^13^C-biomass incorporation by micro-eukaryotes was independent of whichever prey bacteria (Fig. 2B, D), revealing no detectable prey preference in the metabolically active micro-eukaryotic predators. On the contrary, differential labeling by ^13^C-*E. coli* and ^13^C-*P. putida* cells was frequently observed for the predatory bacteria (Fig. 2A, C). The most obvious example was the OM27 clade ASVs belonging to *Bdellovibrionota*, which were found to incorporate ^13^C-labeled biomass exclusively of *E. coli* (Fig. 2C). Comparatively, *Haliangium*-affiliated ASV27 and ASV63 were labeled only by ^13^C-*E. coli*, ASV57 labeled by both ^13^C-*E. coli* and ^13^C-*P. putida*, while ASV72 and ASV76 were also labeled by ^13^C-*P. putida*, but only at a later sampling point (Fig. 2C). These results on the divergent labeling patterns with the tested prey bacteria together strongly implied population-specific predating behaviors of predatory bacteria in activated sludge.

### Diverse Myxococcota and Bdellovibrionota inhabited wastewater treatment plants

The prominent activity of *Myxococcota* and *Bdellovibrionota* species in consuming the added ^13^C-labeled biomass in the microcosm experiment led us to examine their in situ abundance and diversity in activated sludge of local and global WWTPs. First, we profiled activated sludge and anaerobic sludge microbiota in the aeration and anaerobic tanks at a local WWTP (WWTP01) over two years and surprisingly found that *Myxococcota* (6.5 ± 1.3%) accounted for 5.7 times higher relative abundance than *Bdellovibrionota* (1.0 ± 0.2%) in the aerobic activated sludge samples (Fig. 3A). In total, ten myxobacterial genera were found with an average relative sequence abundance >0.1% in the activated sludge of WWTP01, including the putative predators identified in the microcosm experiment, i.e., *Haliangium* spp. (2.8 ± 0.7%) which represented the most abundant myxobacterial lineage in the activated sludge, uncultured *Polyangiaceae* (0.4 ± 0.1%), and the mle1-27 clade (0.2 ± 0.0%; Fig. 3). Moreover, *Pajaroellobacter* (1.2 ± 0.2%), *Nannocystis* (0.4 ± 0.1%), *Phaselicystis* (0.3 ± 0.1%), and several other myxobacterial clades, although not identified as putative predators in the microcosm experiment, were among the abundant myxobacteria in situ in the activated sludge. Although the myxobacterial genera showed comparable relative abundance in the anaerobic tanks, fed by returned activated sludge, to their counterparts in the aerobic tanks, the obligately aerobic myxobacteria were presumably metabolically inactive in the anerobic sludge. Unlike *Myxococcota*, members of *Bdellovibrionota* altogether showed significantly higher relative abundance in the aerobic sludge (1.0 ± 0.2%) than in the anaerobic sludge (0.6 ± 0.1%, paired samples Wilcoxon test *p* < 0.05, *n* = 8) (Fig. 3). The OM27 clade of *Bdellovibrionota* identified as transcriptionally active was abundant predators (0.5 ± 0.2%) in the aerobic activated sludge. *Bdellovibrio* spp. (0.5 ± 0.1%) represented another abundant genus belonging to *Bdellovibrionota*, although it was not enriched by ^13^C-labeled biomass tested in this study.

**Fig. 4:**
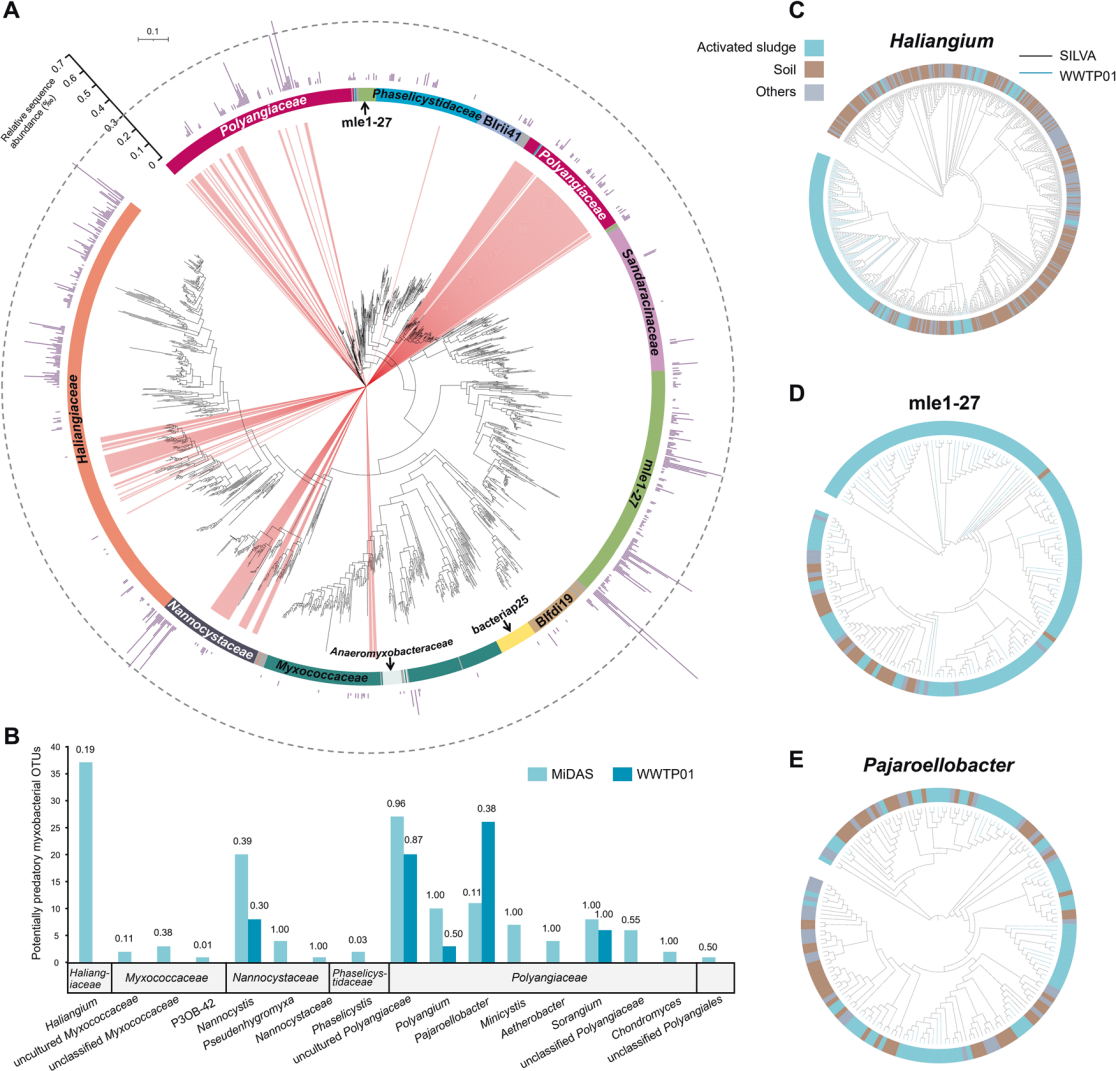
The diversity of *Myxococcota* in global WWTPs. Phylogenetic tree inferred from full-length 16S rRNA gene sequences within the phylum *Myxococcota* originated from global WWTPs (**A**) and the myxobacterial genera with presumed predatory behavior therein (**B**). The tree was constructed with 507 representative OTU sequences from a local WWTP (WWTP01) and 1 010 representative OTU sequences from the MiDAS 4 database [30]. The inner-ring color and labels indicate family classification with the SILVA database [46]. The external bars showed the average relative abundance of the OTUs at the WWTP01 across eight aerobic activated sludge samples collected over two years (maximum 0.07%). The scale bar corresponds to 0.1 substitutions per nucleotide position. Red-colored branches indicate sequences ≥94.5% identical to 16S rRNA gene sequences of myxobacteria with reported predatory behavior (suggesting potential predators at the genus level), the number of which was summarized for each myxobacterial genus in **B**. The numbers above bars indicate the proportion of the potential predators in each genus. Phylogenetic trees were inferred for *Haliangium* (**C**), the mle1-27 clade (**D**), and *Pajaroellobacter* (**E**) across different habitats. The trees were constructed with representative OTU sequences from WWTP01 (174, 134, and 69 sequences, for *Haliangium*, the mle1-27 clade, and *Pajaroellobacter*, respectively) and sequences obtained from the SILVA database [46] with known isolation source (507, 49, and 102 sequences, respectively).

To reveal the diversity of myxobacteria residing in global wastewater treatment systems, we next constructed an integrative myxobacterial 16S rRNA gene dataset by combining the full-length 16S rRNA gene sequences assigned to *Myxococcota* that were generated from the local WWTP01 (507 representative OTU sequences) according to the SILVA taxonomy with sequences from the MiDAS 4 (2 521 sequences clustered to 1 010 OTUs), a well-established 16S rRNA gene reference database for bacteria in WWTPs globally. Phylogenetic analysis showed that *Haliangiaceae* and *Polyangiaceae*, the most diverse [49] and abundant [50] families in soil and other habitats, were well represented in wastewater treatment systems (Fig. 4A). The as-yet-uncultivated mle1-27 clade as well as members of four families, i.e., *Myxococcaceae*, *Sandaracinaceae*, *Nannocystaceae*, and *Phaselicystidaceae*, also contributed significant proportions of the myxobacterial diversity in global WWTPs (Fig. 4A).

The MiDAS and WWTP01 OTUs were classified as potential predators in this study if their full-length 16S rRNA gene sequence showed at least genus-level identity (i.e., ≥94.5%) to those of experimentally verified predatory isolates of *Myxococcota* (none of them from wastewater treatment systems, see a full list in Supplementary Table S2). The identified potential predators in global WWTPs mostly belonged to *Polyangiaceae* (130 out of 209 OTUs), followed by *Haliangiaceae* (37), *Nannocystaceae* (33), *Myxococcaceae* (6), and *Phaselicystidacea* (2) (Fig. 4A, B). Among them, 37 MiDAS OTUs classified as potential predators were taxonomically assigned to *Haliangium*, which was metabolically identified as putative predators in our microcosm experiment, substantiating their important roles in the microbial food webs in global WWTPs. Moreover, all the 173 *Haliangium*-assigned OTUs of WWTP01 were only remotely related to (maximumly 94.3% sequence identity of full-length 16S rRNA gene) myxobacterial predators reported in literature (Fig. 4B), suggesting the existence of possibly novel predatory *Haliangium* species and ecotypes in this local Chinese WWTP. Although members of *Nannocystis*, *Polyangium*, and *Pajaroellobacter* were not enriched by ^13^C-*E. coli* and ^13^C-*P. putida* cell amendment, these three myxobacterial genera were predicted as yet-to-be-verified predators in WWTPs based on full-length 16S rRNA gene sequence identity (94.8–98.1%) to known myxobacterial predators (Fig. 4B). Further phylogenetic analysis applied to myxobacterial 16S rRNA gene sequences from the local WWTP01 combined with sequences from the SILVA database originated from different environments (Fig. 4C–E and Supplementary Fig. S4) revealed that myxobacteria residing in WWTPs tended to form unique clades, phylogenetically distinct from those isolated from soil or other environments, especially for *Haliangium*, mle1-27, and *Nannocystis*, suggesting habitat filtering on myxobacteria in activated sludge.

For *Bdellovibrionota*, *Bdellovibrionaceae* and *Bacteriovoracaceae* species constituted important fractions of their diversity in global WWTPs (310 and 167 out of 1 338 OTUs, respectively; Supplementary Fig. S5), while over half of the collected sequences were assigned to the as-yet-uncultivated 0319-6G20 clade (Supplementary Fig. S5) which warrants future in-depth exploration.

### Myxobacteria were ubiquitous in activated sludge across global wastewater treatment plants

**Fig. 5:**
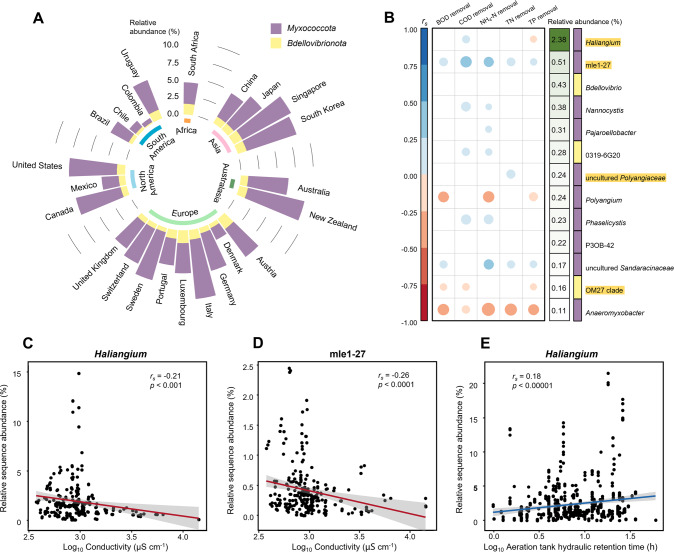
Global distribution and functional potentials of *Myxococcota* and *Bdellovibrionota* in activated sludge. The analysis was performed based on microbiota datasets of 1 186 activated sludge samples from 269 WWTPs globally published by the Global Water Microbiome Consortium [2]. Stacked bars (**A**) showed the average relative sequence abundance of *Myxococcota* and *Bdellovibrionota* across the samples collected from each country. The potential impact of predatory bacteria on sludge performance was assessed by testing the correlation (**B**) between their abundance and the removal of BOD (biochemical oxygen demand), COD (chemical oxygen demand), ammonia nitrogen (NH_4_-N), total nitrogen (TN), and total phosphorus (TP). The color and size of the circles indicate Spearman’s rank correlation coefficients, and circles were displayed only for significant correlation (*p* < 0.05, *n* = 529, 281, 423, 268, and 364, for BOD, COD, NH_4_-N, TN, and TP removal, respectively, corrected with the Benjamini–Hochberg method for multiple testing). Only the genus-level taxa with average relative sequence abundance >0.1% across all the 1 186 samples are shown, with the putative predators identified by SIP in the microcosm experiment marked with a yellow background. Genera of *Myxococcota* and *Bdellovibrionota* are indicated with purple and yellow bars, respectively. Examples showed how the sludge properties potentially influence *Myxococcota*, including that conductivity was negatively correlated with the relative sequence abundance of *Haliangium* (**C**) and mle1-27 (**D**), whereas aeration tank hydraulic retention time showed positive correlation with *Haliangium* (**E**).

To depict the biogeographic distribution patterns of predatory bacteria (e.g., members of *Myxococcota* and *Bdellovibrionota*) and explore their ecological roles and environmental drivers in activated sludge on a global scale, we exploited a 16S rRNA gene amplicon dataset of 1 186 activated sludge samples from 269 WWTPs worldwide published by the Global Water Microbiome Consortium [2]. To facilitate effective cross-study comparison, bacterial taxonomy was re-assigned to the representative sequences of OTUs using the SILVA SSU database (version 138), following the same bioinformatics procedures and cutoffs as applied in our study. Overall, we found that myxobacteria were ubiquitously detected (in 1 183 samples) in activated sludge of global WWTPs, constituting a non-negligible proportion (5.4 ± 0.1%) of activated sludge microbiota, and being consistently more abundant than *Bdellovibrionota* (1.1 ± 0.0%; Fig. 5A). While both *Myxococcota* and *Bdellovibrionota* lineages showed the lowest average relative abundance in the South America (2.7 ± 0.3% and 0.7 ± 0.1%, respectively; *n* = 80), they were the most abundant in North America (6.5 ± 0.2%, *n* = 616) and Africa (1.5 ± 0.1%, *n* = 36), respectively, suggesting a geographic distribution (*p* < 10^−10^ for both, Kruskal–Wallis test; Fig. 5A). Similar to the observation at the local WWTP, *Haliangium*, members of which were identified among the main putative predators in microcosm experiment, was the most abundant genus of *Myxococcota*, accounting for an average relative abundance of 2.4 (±0.1) % in the global activated sludge samples, followed by the mle1-27 clade (0.5%), *Nannocystis* (0.4%), and *Pajaroellobacter* (0.3%) (Fig. 5B). Comparatively, *Bdellovibrio* was the most abundant genus of *Bdellovibrionota*, with an average relative abundance of 0.4% (Fig. 5B).

To discern the potential impacts of predatory bacteria on activated sludge process performance, Spearman’s rank correlation analysis was performed to identify significant associations (FDR-adjusted *p* < 0.05) between removal rates of organic carbon (BOD and COD) and nutrient (NH_4_-N, TN and TP) pollutants and relative abundance of *Myxococcota* and *Bdellovibrionota* genera in global WWTPs by re-analyzing performance data provided by the Global Water Microbiome Consortium [2]. *Haliangium* spp. were found to significantly correlate positively with COD removal (*r*_*s*_ = 0.16), but negatively correlate with total phosphorus (TP) removal (*r*_*s*_ = −0.12; Fig. 5B). The second most abundant myxobacterial mle1-27 clade displayed significantly positive correlation with all the investigated removal parameters (*r*_*s*_: 0.13 to 0.31). Consistently, the mle1-27 clade correlated positively with *Tetrasphaera* (*r*_*s*_ = 0.18; Supplementary Fig. S6), a polyphosphate-accumulating organism, and a variety of denitrifying bacteria, including *Dechloromonas*, *Zoogloea*, *Thauera*, *Comamonas* (*r*_*s*_: 0.12–0.17), and especially *Candidatus* Accumulibacter (*r*_*s*_ = 0.39; Supplementary Fig. S6). On the contrary, the removal of BOD, COD, and TP was negatively related with the OM27 clade (*r*_*s*_: −0.13 to −0.12) and *Anaeromyxobacter* (*r*_*s*_: −0.17 to −0.33) belonging to *Bdellovibrionota* and *Myxococcota*, respectively (Fig. 5B). In addition, no significant correlation was observed between *Bdellovibrio* spp. and the investigated pollutant removal rates.

Further correlation analysis revealed the differential effects imposed by activated sludge process parameters on members of *Myxococcota* and *Bdellovibrionota*. The *Myxococcota* and *Bdellovibrionota* lineages could mostly benefit from longer hydraulic retention time (Fig. 5E and Supplementary Fig. S7), whereas higher pH restrained most of these bacteria (Supplementary Fig. S7). Conductivity was negatively correlated with *Haliangium* (Fig. 5C), the mle1-27 clade (Fig. 5D), *Pajaroellobacter*, and the 0319-6G20 clade (*r*_*s*_ < −0.1, all adjusted *p* < 0.001; Supplementary Fig. S4), but was positively correlated with *Nannocystis* (*r*_*s*_ = 0.17, adjusted *p* < 0.01; Supplementary Fig. S7).

## Discussion

Predatory bacteria are key components of activated sludge microbiomes in the wastewater treatment systems of critical ecological and functional importance, but their predatory activity, phylogenetic diversity, and geographic distribution were almost unknown. In this study, we combined rRNA-SIP using ^13^C-labeled bacterial prey in microcosms with long-term microbiota monitoring at a local WWTP and reanalysis of global WWTP microbiomes to jointly uncovered the overlooked diversity of predatory bacteria in activated sludge, especially for myxobacteria, gaining first and unique insights into a considerable proportion of as-yet-cultivated predatory bacterial diversity in the activated sludge of both local and global WWTPs, highlighting their potential links with wastewater treatment processes through removal of undesired enteric bacteria and regulation of functional ones.

### Myxobacteria as predominantly active predatory bacteria in activated sludge

Prior research characterizing predatory bacteria in WWTPs were almost exclusively focused on BALOs, which demonstrated their role in regulation of microbial communities [26] and the application potential in facilitating waste sludge dewaterability [27] of a few isolates of BALOs [27, 51]. These culture-based investigations delivered insightful understanding of the isolates but left the vast diversity of active predatory bacteria (mostly as-yet-uncultivated) in WWTPs largely unexplored. By virtue of SIP with ^13^C-labeled prey cells, we were able to substantially expand the list of predatory bacteria in this specific ecosystem and uncover the surprisingly high activity of predatory myxobacteria in activated sludge, in agreement with our first hypothesis that predatory bacteria dominated by myxobacteria could play active roles in microbial trophic network in WWTPs. The higher diversity of ^13^C-biomass incorporators belonging to *Myxococcota* than *Bdellovibrionota* at both genus (Fig. 2A) and ASV (Fig. 2C) levels in the microcosm experiment, the larger proportion of rRNA transcripts made up by *Myxococcota* groups (Fig. 1D, E), together with their consistently higher abundance in the local WWTP (Fig. 3) and across the global WWTPs (Fig. 5A), strongly indicated the dominance of *Myxococcota* species in the active predatory bacteria of WWTPs. In fact, members of *Myxococcota* were frequently detected in both amplicon and metagenomic sequencing-based studies of activated sludge microbiomes [2, 28, 30, 52], but attempts to disentangle their functional importance in WWTPs were missing. This study applied the culture-independent SIP approach to obtain the first direct evidence of in situ intra-bacterial predation predominated by myxobacteria in WWTPs. To our surprise, *Bdellovibrionota* was comparatively much less represented in the predatory prokaryotes consuming the ^13^C-labeled biomass (Fig. 2A), which we believe should not be attributed to the limited number of prey bacteria utilized in this study, as *E. coli* and *Pseudomonas* species are among the favorable prey for BALOs across various environments [53]. It is worth mentioning that isotopic labeling through cross-feeding could not be completely excluded for this adaptation of SIP, which makes the labeling mechanisms of other bacteria than *Myxococcota* and *Bdellovibrionota* lineages have to be interpreted cautiously. Nevertheless, the prompt, predominant, and consistent labeling of myxobacteria as revealed by time-resolved sampling efforts of this study served as direct evidence for their predating activity in activated sludge.

Two myxobacterial groups, i.e., *Haliangium* spp. and the mle1-27 clade were identified as the most important putative bacterial predators according to their strong isotopic labeling in the microcosms (Fig. 2A) and in situ prevalence in global WWTPs (Figs. 3 and 5B). Our study has demonstrated for the first time the as-yet-uncultivated *Haliangium* spp. as predatorily active and the most abundant myxobacteria in activated sludge, whether in the local WWTP over time or across the global WWTPs examined, clearly pointing to their important trophic roles in these engineering ecosystems. With the first culturable representatives isolated from coastal environment [54], *Haliangium* spp. has also exhibited notable predating activity in an agricultural soil [40]. Specifically, a *Haliangium* OTU, which showed 98.8% identity to our experimentally validated predatory ASV27 (Fig. 2C), was among the 28 OTUs identified as the global core activated sludge members [2], the functional role of which as bacterial predators ubiquitous in global WWTPs could be homologically inferred from this study. In addition, all of the *Haliangium* OTUs from the local WWTP were only remotely related to (maximumly 94.3% sequence identity of full-length 16S rRNA gene) reported predatory isolates of myxobacteria (Fig. 4B), suggesting the existence of novel predatory *Haliangium* species in the local WWTP. Similarly, the as-yet-uncultivated mle1-27 clade was phylogenetically distant from all the known predatory myxobacteria in literature (Fig. 4A), although they showed intensive incorporation of the ^13^C-labeled biomass. Furthermore, activated sludge represents the major habitat for the myxobacterial mle1-27 clade (Fig. 4D). The above results together expand our current understanding of predatory bacteria in activated sludge by revealing the prevalence of as-yet-uncultivated but highly active myxobacterial predators in WWTPs which are worth further targeted investigation.

The myxobacteria in global WWTPs mostly belonged to *Haliangiaceae*, *Polyangiaceae*, the mle1-27 clade, *Myxococcaceae*, *Sandaracinaceae*, *Nannocystaceae*, and *Phaselicystidaceae*, with the former two families also the most diverse [49] and abundant [50] families across different environments. Myxobacterial taxa are known to show differential preference for environmental types [31, 49]. The phylogenetic affinity within most *Myxococcota* species from WWTPs suggested habitat filtering in the global wastewater treatment systems. Genomics- and metagenomics-based efforts [55] are demanded to shed light on the metabolic features of myxobacteria in activated sludge, and mechanisms underlying the diversity patterns of predatory bacteria in WWTPs and across other environments.

### Divergent predation behaviors between predatory bacteria and protists

SIP-based investigation of microbial predation in activated sludge also enables elucidation of inter-domain and intra-genus divergent predatory behaviors. Besides predatory bacteria, a variety of micro-eukaryotes dominated by members of *Ciliophora*, especially *Cyrtophoria* spp. and *Telotrochidium* spp., were also identified to actively incorporate the ^13^C-labeled prey biomass (Fig. 3B, D). Ciliates have since long been regarded as the important protozoan predators in activated sludge [56]. Moreover, our results suggest that the ciliates predate irrespective of the prey species provided in this study, whereas members of *Myxococcota* and *Bdellovibrionota* largely showed prey preference for *E. coli* over *P. putida* (Fig. 2), supporting our second hypothesis that predatory bacteria are prey-selective, unlike protists, in such a complex engineered ecosystem. Protists are generally considered to adopt a generalist predating strategy, while predatory bacteria tend to be more selective [7]. For instance, myxobacteria typically predate preferentially on Gram-negative over Gram-positive bacteria [40, 57]. Here, our SIP-based investigation of microbial trophic interactions provides valuable in situ evidence on the inter-domain predation behavior divergence and selective predation by predatory bacteria in activated sludge, which adds to the feasibility of applying predatory myxobacteria for targeted trophic regulations of activated sludge microbiomes, and eventually process performance. Specifically, some *Haliangium* ASVs and the OM27 clade preyed exclusively on *E. coli* (Fig. 2C), indicating the differentiated predation strategies between different predatory bacteria and even within the same genus, i.e., *Haliangium*. The feeding preference revealed for predatory bacteria might also explain the absence of potential predators (e.g., *Nannocystis*, *Polyangium*, and *Pajaroellobacter*) detected based on 16S rRNA gene sequence identity to known predators in the ^13^C-biomass incorporators, due to the limited number of prey species used in this study that fall beyond the prey range of these potential predators. Future experiments with a wider variety of bacterial prey species could reveal a larger diversity of microbial predators in activated sludge. Nevertheless, our findings demonstrated the power of SIP in resolving microbial food web ecological patterns, e.g., competitive trophic interactions, in complex environments.

### Environmental implications of bacterial predation on WWTP functioning

In accordance with the selective predation feature of *Haliangium* spp., their abundance positively correlated with COD removal rate and negatively with total phosphorus removal rate across the global WWTPs, whereas the mle1-27 clade that displayed no prey preference showed positive correlation with all the pollutant removal rates tested (Fig. 5B) as well as with functional bacteria such as polyphosphate-accumulating and denitrifying bacteria (Supplementary Fig. S6). In contrast, *Bdellovibrio* spp. appeared not related to activated sludge performance across global WWTPs, agreeing with a previous report that predation by *B. bacteriovorus* alters community composition but not functional profiles of activated sludge microbiota [26]. Top-down control on microbiota by predators work via both direct effect on prey and indirect resource-driven effect. Accordingly, it can be speculated in wastewater treatment systems that the selective predation by predatory bacteria could eliminate undesired bacteria (e.g., *E. coli* as demonstrated in this study), and in other cases promoted the growth of specific pollutant-removing bacteria, for example, by releasing their competitive pressure [58, 59], and therefore accelerated the relevant nutrient removal processes. Thus, selective predation serves as the basis for targeted regulation of microbiota and the functioning processes they govern. In particular, among the array of activated sludge process parameters examined, hydraulic retention time was found to positively correlate with the abundance of predatory bacteria, while high pH showed negative impacts. Such knowledge could help improve enrichment and isolation outcomes for different predatory bacteria. Salinity was proposed as an important influencing factor for the global geographic distribution and diversity of myxobacteria [49]. Similarly, our study showed that higher conductivity in activated sludge favored some myxobacterial genera, including *Haliangium* spp. and the mle1-27 clade, whereas restrained *Nannocystis* spp., uncultured *Sandaracinaceae*, and *Anaeromyxobacter* species.

By combining multiple lines of evidence from microcosm isotope labeling, phylogenetic, and global prevalence analyses, this study sheds light on the diversity and metabolic activities of predatory bacteria in activated sludge, discovers the previously overlooked key members of the microbial food webs therein, and provides first hints on how they could interact with other core constituents and functional microbes in activated sludge. Considering the worldwide prevalence, trophic function, and huge as-yet-uncultivated fraction of *Myxococcota* species in activated sludge, they deserve more attention to achieve the discovery of novel myxobacterial resources, an advanced understanding of their ecological roles in activated sludge microbiota, and targeted regulation and management of biological wastewater treatment processes based on predator-prey relationships. Future efforts in retrieving and analyzing high-quality myxobacterial genomes from activated sludge systems based on, for example, metagenomic or single-cell genomic analysis, should provide new knowledge on their ecophysiology and metabolism to guide strain cultivation, which is essential for biocontrol application of such eco-friendly predatory bacteria in wastewater treatment systems via trophic regulation.

## Supplementary information

Supplementary materials

## Data Availability

The raw sequencing data generated during the current study have been deposited in the NCBI’s SRA database (SRP408186) and CNGB Sequence Archive (CNSA) of China National GeneBank DataBase (CNP0003718). Additional sequences analyzed during the current study are publicly available from the SILVA (https://www.arb-silva.de) and MIDAS 4 (https://www.midasfieldguide.org/guide) databases. The global activated sludge microbiome dataset re-analyzed in this study is available from the Global Water Microbiome Consortium (http://gwmc.ou.edu/data-disclose.html).

## References

[1] Ju F, Zhang T (2015). Bacterial assembly and temporal dynamics in activated sludge of a full-scale municipal wastewater treatment plant. ISME J.

[2] Wu L, Ning D, Zhang B, Li Y, Zhang P, Shan X (2019). Global diversity and biogeography of bacterial communities in wastewater treatment plants. Nat Microbiol.

[3] Ju F, Xia Y, Guo F, Wang Z, Zhang T (2014). Taxonomic relatedness shapes bacterial assembly in activated sludge of globally distributed wastewater treatment plants. Environ Microbiol.

[4] Singleton CM, Petriglieri F, Kristensen JM, Kirkegaard RH, Michaelsen TY, Andersen MH (2021). Connecting structure to function with the recovery of over 1000 high-quality metagenome-assembled genomes from activated sludge using long-read sequencing. Nat Commun.

[5] Ratsak CH, Maarsen KA, Kooijman SALM (1996). Effects of protozoa on carbon mineralization in activated sludge. Water Res.

[6] Al-Shahwani SM, Horan NJ (1991). The use of protozoa to indicate changes in the performance of activated sludge plants. Water Res.

[7] Johnke J, Cohen Y, de Leeuw M, Kushmaro A, Jurkevitch E, Chatzinotas A (2014). Multiple micro-predators controlling bacterial communities in the environment. Curr Opin Biotechnol.

[8] Pérez J, Moraleda-Muñoz A, Marcos-Torres FJ, Muñoz-Dorado J (2016). Bacterial predation: 75 years and counting!. Environ Microbiol.

[9] Sockett RE, Lambert C (2004). *Bdellovibrio* as therapeutic agents: a predatory renaissance?. Nat Rev Microbiol.

[10] Kadouri DE, To K, Shanks RMQ, Doi Y (2013). Predatory bacteria: a potential ally against multidrug-resistant Gram-negative pathogens. PLOS One.

[11] Burian A, Pinn D, Peralta-Maraver I, Sweet M, Mauvisseau Q, Eyice O (2021). Predation increases multiple components of microbial diversity in activated sludge communities. ISME J.

[12] Freudenthal J, Ju F, Bürgmann H, Dumack K (2022). Microeukaryotic gut parasites in wastewater treatment plants: diversity, activity, and removal. Microbiome.

[13] Shapiro OH, Kushmaro A, Brenner A (2010). Bacteriophage predation regulates microbial abundance and diversity in a full-scale bioreactor treating industrial wastewater. ISME J.

[14] Chen Y, Wang Y, Paez-Espino D, Polz MF, Zhang T (2021). Prokaryotic viruses impact functional microorganisms in nutrient removal and carbon cycle in wastewater treatment plants. Nat Commun.

[15] Sockett RE (2009). Predatory lifestyle of *Bdellovibrio bacteriovorus*. Annu Rev Microbiol.

[16] Guerrero R, Pedros-Alio C, Esteve I, Mas J, Chase D, Margulis L (1986). Predatory prokaryotes: predation and primary consumption evolved in bacteria. Proc Natl Acad Sci USA.

[17] Wang Z, Kadouri DE, Wu M (2011). Genomic insights into an obligate epibiotic bacterial predator: *Micavibrio aeruginosavorus* ARL-13. BMC Genom.

[18] Wang W, Luo X, Ye X, Chen Y, Wang H, Wang L (2020). Predatory Myxococcales are widely distributed in and closely correlated with the bacterial community structure of agricultural land. Appl Soil Ecol.

[19] Chauhan A, Cherrier J, Williams HN (2009). Impact of sideways and bottom-up control factors on bacterial community succession over a tidal cycle. Proc Natl Acad Sci USA.

[20] Ye X, Li Z, Luo X, Wang W, Li Y, Li R (2020). A predatory myxobacterium controls cucumber Fusarium wilt by regulating the soil microbial community. Microbiome.

[21] Zhao Y-J, Sato Y, Inaba T, Aoyagi T, Hori T, Habe H (2019). Activated sludge microbial communities of a chemical plant wastewater treatment facility with high-strength bromide ions and aromatic substances. J Gen Appl Microbiol.

[22] Waite DW, Chuvochina M, Pelikan C, Parks DH, Yilmaz P, Wagner M (2020). Proposal to reclassify the proteobacterial classes *Deltaproteobacteria* and *Oligoflexia*, and the phylum *Thermodesulfobacteria* into four phyla reflecting major functional capabilities. Int J Syst Evol Microbiol.

[23] Rendulic S, Jagtap P, Rosinus A, Eppinger M, Baar C, Lanz C (2004). A predator unmasked: life cycle of *Bdellovibrio bacteriovorus* from a genomic perspective. Science.

[24] Mohr KI (2018). Diversity of myxobacteria-we only see the tip of the iceberg. Microorganisms..

[25] Zhou XW, Li SG, Li W, Jiang DM, Han K, Wu ZH (2014). Myxobacterial community is a predominant and highly diverse bacterial group in soil niches. Environ Microbiol Rep.

[26] Feng S, Tan CH, Constancias F, Kohli GS, Cohen Y, Rice SA (2017). Predation by *Bdellovibrio bacteriovorus* significantly reduces viability and alters the microbial community composition of activated sludge flocs and granules. FEMS Microbiol Ecol.

[27] Yu R, Zhang S, Chen Z, Li C (2017). Isolation and application of predatory *Bdellovibrio*-and-like organisms for municipal waste sludge biolysis and dewaterability enhancement. Front Environ Sci Eng.

[28] Ye L, Mei R, Liu W-T, Ren H, Zhang X-X (2020). Machine learning-aided analyses of thousands of draft genomes reveal specific features of activated sludge processes. Microbiome.

[29] Yuan L, Wang Y, Zhang L, Palomo A, Zhou J, Smets BF (2021). Pathogenic and indigenous denitrifying bacteria are transcriptionally active and key multi-antibiotic-resistant players in wastewater treatment plants. Environ Sci Technol.

[30] Dueholm MKD, Nierychlo M, Andersen KS, Rudkjøbing V, Knutsson S, Arriaga S (2022). MiDAS 4: A global catalogue of full-length 16S rRNA gene sequences and taxonomy for studies of bacterial communities in wastewater treatment plants. Nat Commun.

[31] Liu Y, Yao Q, Zhu H (2019). Meta-16S rRNA gene phylogenetic reconstruction reveals the astonishing diversity of cosmopolitan myxobacteria. Microorganisms..

[32] Radajewski S, Ineson P, Parekh NR, Murrell JC (2000). Stable-isotope probing as a tool in microbial ecology. Nature..

[33] Winderl C, Penning H, von Netzer F, Meckenstock RU, Lueders T (2010). DNA-SIP identifies sulfate-reducing *Clostridia* as important toluene degraders in tar-oil-contaminated aquifer sediment. ISME J..

[34] Orsi WD, Smith JM, Liu S, Liu Z, Sakamoto CM, Wilken S (2016). Diverse, uncultivated bacteria and archaea underlying the cycling of dissolved protein in the ocean. ISME J.

[35] Ziels R, Sousa D, Stensel HD, Beck D (2017). DNA-SIP based genome-centric metagenomics identifies key long-chain fatty acid-degrading populations in anaerobic digesters with different feeding frequencies. ISME J.

[36] Costa OYA, de Hollander M, Pijl A, Liu B, Kuramae EE (2020). Cultivation-independent and cultivation-dependent metagenomes reveal genetic and enzymatic potential of microbial community involved in the degradation of a complex microbial polymer. Microbiome..

[37] Orsi WD, Wilken S, del Campo J, Heger T, James E, Richards TA (2018). Identifying protist consumers of photosynthetic picoeukaryotes in the surface ocean using stable isotope probing. Environ Microbiol.

[38] Lueders T, Kindler R, Miltner A, Friedrich MW, Kaestner M (2006). Identification of bacterial micropredators distinctively active in a soil microbial food web. Appl Environ Microbiol.

[39] Moreno AM, Matz C, Kjelleberg S, Manefield M (2010). Identification of ciliate grazers of autotrophic bacteria in ammonia-oxidizing activated sludge by RNA stable isotope probing. Appl Environ Microbiol.

[40] Zhang L, Lueders T (2017). Micropredator niche differentiation between bulk soil and rhizosphere of an agricultural soil depends on bacterial prey. FEMS Microbiol Ecol.

[41] Haig SJ, Schirmer M, D’Amore R, Gibbs J, Davies RL, Collins G (2015). Stable-isotope probing and metagenomics reveal predation by protozoa drives *E. coli* removal in slow sand filters. ISME J.

[42] Lueders T, Dumont MG, Bradford L, Manefield M (2016). RNA-stable isotope probing: from carbon flow within key microbiota to targeted transcriptomes. Curr Opin Biotechnol.

[43] Zhang Z, Zhang G, Ju F (2022). Using culture-enriched phenotypic metagenomics for targeted high-throughput monitoring of the clinically important fraction of the beta-lactam resistome. Environ Sci Technol.

[44] Lueders T. DNA- and RNA-based stable isotope probing of hydrocarbon degraders. In: McGenity TJ, Timmis KN, Nogales B, editors. Hydrocarbon and lipid microbiology protocols: genetic, genomic and system analyses of communities. Springer Protocols Handbooks. Berlin, Heidelberg: Springer; 2015, 181–97.

[45] Bolyen E, Rideout JR, Dillon MR, Bokulich NA, Abnet CC, Al-Ghalith GA (2019). Reproducible, interactive, scalable and extensible microbiome data science using QIIME 2. Nat Biotechnol.

[46] Callahan BJ, McMurdie PJ, Rosen MJ, Han AW, Johnson AJA, Holmes SP (2016). DADA2: High-resolution sample inference from Illumina amplicon data. Nat Methods.

[47] Quast C, Pruesse E, Yilmaz P, Gerken J, Schweer T, Yarza P (2013). The SILVA ribosomal RNA gene database project: improved data processing and web-based tools. Nucleic Acids Res.

[48] Kramer S, Dibbern D, Moll J, Huenninghaus M, Koller R, Krueger D (2016). Resource partitioning between bacteria, fungi, and protists in the detritusphere of an agricultural soil. Front Microbiol.

[49] Wang J, Wang J, Wu S, Zhang Z, Li Y (2021). Global geographic diversity and distribution of the myxobacteria. Microbiol Spectr.

[50] Petters S, Gross V, Sollinger A, Pichler M, Reinhard A, Bengtsson MM (2021). The soil microbial food web revisited: Predatory myxobacteria as keystone taxa?. ISME J.

[51] Feng S, Tan CH, Cohen Y, Rice SA (2016). Isolation of *Bdellovibrio bacteriovorus* from a tropical wastewater treatment plant and predation of mixed species biofilms assembled by the native community members. Environ Microbiol.

[52] Wang Y, Ye J, Ju F, Liu L, Boyd JA, Deng Y (2021). Successional dynamics and alternative stable states in a saline activated sludge microbial community over 9 years. Microbiome.

[53] Williams HN, Chen H (2020). Environmental regulation of the distribution and ecology of *Bdellovibrio* and like organisms. Front Microbiol.

[54] Fudou R, Jojima Y, Iizuka T, Yamanaka S (2002). *Haliangium ochraceum* gen. nov., sp. nov. and *Haliangium tepidum* sp. nov.: Novel moderately halophilic myxobacteria isolated from coastal saline environments. J Gen Appl Microbiol.

[55] Murphy CL, Yang R, Decker T, Cavalliere C, Andreev V, Bircher N (2021). Genomes of novel *Myxococcota* reveal severely curtailed machineries for predation and cellular differentiation. Appl Environ Microbiol.

[56] Esteban G, Téllez C, Bautista LM (1991). Dynamics of ciliated protozoa communities in activated sludge process. Water Res.

[57] Morgan AD, MacLean RC, Hillesland KL, Velicer GJ (2010). Comparative analysis of *Myxococcus* predation on soil bacteria. Appl Environ Microbiol.

[58] Terborgh JW (2015). Toward a trophic theory of species diversity. Proc Natl Acad Sci USA.

[59] Thakur MP, Geisen S (2019). Trophic regulations of the soil microbiome. Trends Microbiol.

