# Supplementary material for: Active predation, phylogenetic diversity, and global prevalence of myxobacteria in wastewater treatment plants
Source: ISME J. 2023 Feb 11;17(5):671–81. doi: 10.1038/s41396-023-01378-0 (PMC9919749; doi:10.1038/s41396-023-01378-0)
Supplement: Supplementary file 1 — Supplementary materials [file 41396_2023_1378_MOESM1_ESM.docx]

**SUPPLEMENTARY MATERIAL for**

Active Predation, Diversity, and Global Prevalence of Myxobacteria in Wastewater Treatment Plants

Lu Zhang^1,2,3^, Xinyu Huang^1,2,3^, Jizhong Zhou^4^, and Feng Ju^1,2,4,5*^

^1^ Research Center for Industries of the Future, Key Laboratory of Coastal Environment and Resources of Zhejiang Province, School of Engineering, Westlake University, Hangzhou, Zhejiang Province, China

^2^ Center of Synthetic Biology and Integrated Bioengineering, School of Engineering, Westlake University, Hangzhou, Zhejiang Province, China

^3^ Institute of Advanced Technology, Westlake Institute for Advanced Study, Hangzhou, Zhejiang Province, China

^4^ Institute for Environmental Genomics, Department of Microbiology and Plant Biology, University of Oklahoma, Norman, Oklahoma, United States

^5^ Westlake Laboratory of Life Sciences and Biomedicine, Hangzhou, Zhejiang Province, China

***Corresponding author contact:** Tel.: 571-87963205 (lab), 571-87380995 (office), E-mail: [jufeng@westlake.edu.cn](mailto:jufeng@westlake.edu.cn)

**Supplementary Methods**

**Method S1** Sludge sampling

**Method S2** ^13^C-labelling of prey bacteria

**Method S3** Microcosm incubation

**Method S4** ^13^CO_2_ measurement

**Method S5** RNA extraction and gradient centrifugation

**Method S6** Quantitative reverse transcription PCR

**Method S7** Fraction sequencing and sequence analysis

**Method S8** WWTP sludge DNA extraction, sequencing, and data analyses

**Method S9** Global WWTP full-length bacterial 16S rRNA gene sequences and phylogenetic analyses

**Method S10** Global activated sludge microbiome dataset processing and statistical analyses

**Figure and Table Legends**

**Fig. S1** Copies of 16S rRNA gene transcripts across the entire buoyant density gradient of the RNA extracted from activated sludge microcosms amended with *E. coli* and *P. putida* cells after one, two, and four days of incubation. The 16S rRNA gene transcripts were quantified using real-time reverse transcription PCR. The rRNA fractions marked with grey background were subjected to the subsequent amplicon sequencing analysis.

**Fig. S2** The dynamics of the micro-eukaryotic communities during the microcosm experiment. Structure of the active micro-eukaryotic communities was inferred based on amplicon sequencing of the light rRNA fractions from the microcosms amended with ^13^C-*E. coli* (A) and ^13^C-*P. putida* (B) cells. The overall micro-eukaryotic communities were obtained by 18S rRNA gene amplicon sequencing of the total DNA from the activated sludge microcosms amended with ^13^C-*E. coli* (C) and ^13^C-*P. putida* (D) cells, and the control group (E) without amendment. Relative sequence abundance of the ten most abundant micro-eukaryotic phyla was shown.

**Fig. S3** Taxonomic diversity of the ^13^C-incorporators identified by SIP. Labelled prokaryotic (A) and micro-eukaryotic (B) ASVs were determined with an EF threshold > 0.1, and only ASVs with relative sequence abundance > 0.1% in the heavy rRNA fractions of at least one of the ^13^C-*E. coli* and ^13^C-*P. putida* microcosms at a sampling point were counted. The labelled proteobacterial ASVs were exclusively affiliated with *Escherichia-Shigella* or *Pseudomonas* (E/P).

**Fig. S4** Phylogenetic trees inferred for selected myxobacterial genera from near full-length 16S rRNA gene sequences across different habitats. The trees were constructed with representative OTU sequences from a local WWTP (174, 134, 69, 27, and 6 sequences, for *Haliangium*, the mle1-27 clade, *Pajaroellobacter*, *Nannocystis*, and *Polyangium*, respectively) and sequences obtained from the SILVA database with known isolation source (507, 49, 102, 38, and 10 sequences, respectively).

**Fig. S5** Phylogenetic tree inferred from full-length 16S rRNA gene sequences within the phylum *Bdellovibrionota* originated from global WWTPs. The tree was constructed with 105 representative OTU sequences from a local WWTP (WWTP01) and 1 233 reference sequences from the MiDAS 4 database. The external bars showed average relative abundance of the OTUs at the WWTP01 across eight aerobic activated sludge samples collected over two years (maximum 0.014%). The scale bar corresponds to 0.1 substitutions per nucleotide position.

**Fig. S6** The correlation between members of *Myxococcota* and *Bdellovibrionota* and sludge performance-relevant bacteria in global activated sludge, evaluated using data acquired from the Global Water Microbiome Consortium of 1 186 activated sludge samples from 269 WWTPs across 23 countries. Color and size of the circles indicate Spearman’s rank correlation coefficients, and circles were displayed only for significant correlation (*p* < 0.05, n = 1 186). Correlation among only selected *Myxococcota* and *Bdellovibrionota* genus-level taxa, as well as sludge performance-relevant bacteria with average relative sequence abundance > 0.1% across all the 1 186 samples were analyzed and displayed. AOB, ammonia-oxidizing bacteria; DNB, denitrifying bacteria; PAO, polyphosphate-accumulating organism; GAO, glycogen-accumulating organism.

**Fig. S7** The impact of sludge process parameters on predatory bacteria in activated sludge, evaluated using data acquired from the Global Water Microbiome Consortium of 1 186 activated sludge samples worldwide. Color and size of the circles indicate Spearman’s rank correlation coefficients, and circles were displayed only for significant correlation (*p* < 0.05, n = 634, 844, 765, 927, 869, 787, and 248, for HRT, SRT, MLSS, DO, pH, temperature, and conductivity, respectively, corrected with the Benjamini-Hochberg method for multiple testing). Only the genus-level taxa with average relative sequence abundance > 0.1% across all the 1 186 samples are shown, with the putative predators identified by SIP in microcosm experiment marked with grey background. HRT, aeration tank hydraulic retention time; SRT, solid retention time; MLSS, mixed liquor suspended solids; DO, dissolved oxygen.

**Table S1** Sampling dates from March 2019 to April 2021. Sludge samples were collected from the aeration and anaerobic tanks at the same sampling points.

**Table S2** Source information of the 129 collected sequences of *Myxococcota* isolates with reported experimental evidence of predatory capability.

# Supplementary Methods

## Method S1 Sludge sampling

The sludge samples used in this study were collected from a local municipal WWTP in Hangzhou, China, which uses an anaerobic/anoxic/oxic (A^2^/O) process. Sludge samples for microbiome monitoring were obtained from the aeration and anaerobic tanks from March 2019 to April 2021 (eight samplings, Table S1) at the same sampling points. The activated sludge used in the microcosm experiment was sampled from an aeration tank in May 2021, transported to the laboratory in a cooling box (~4 °C) within one hour, before the sludge microcosms were set up immediately. The total solids and volatile solids were 6.02±0.72 g L^-1^ and 3.31±0.37 g L^-1^, respectively.

## Method S2 ^13^C-labelling of prey bacteria

Two bacterial strains i.e., *Escherichia coli* ESS5 and *Pseudomonas putida* ESE1, were used as prey bacteria in the SIP microcosm experiment. The strains were isolated from the sampled WWTP and identified by MALDI-TOF mass spectrometry [1]. To achieve ^13^C-labelling, bacteria were grown in minimal salt medium (MSM; Coolaber) containing 4 g l^-1^ 99% ^13^C_6_-glucose (Cambridge Isotope Laboratories, Inc.) as sole carbon source. The same strains were cultivated in MSM medium with ^12^C-glucose (Sigma Aldrich). Bacterial cells were grown for ~20 h followed by a transfer with the same medium, before harvested by centrifugation at the speed of 10 000 g and 4 °C for 10 min, five times of washing, and final resuspension in 50 ml glucose-free MSM. A 100-μl aliquot of each cell suspension was taken and diluted to determine cell concentration using a hemocytometer. ^13^C-labelling of the harvested bacterial cells was determined by a gas isotope ratio mass spectrometer (IRMS; MAT 271, Thermo Scientific) at Shanghai Research Institute of Chemical Industry. Lyophilized cells were combusted at 800 °C in a muffle furnace for 4 h in the presence of Cu_2_O, and the atom percentage of ^13^CO_2_ in the generated CO_2_ was measured using IRMS.

## Method S3 Microcosm incubation

The ^13^C-labelled *E. coli* and *P. putida* cells in 0.5 ml volume were added to 10 ml of activated sludge to achieve a targeted concentration of 2 ╳ 10^8^ cells ml^-1^ in glass bottles closed with screw cap and rubber stopper. In parallel, ^12^C-*E. coli* and *P. putida* cells were added to the ^12^C-microcosms and a control group without cell amendment was set up. Bottles were destructively sampled with duplicate microcosms after 16 hours, one day, two days, four days, and eight days of incubation, resulting in a total of 50 glass bottles. All the microcosms were started at the same time, and constructed with 125-ml glass bottles, except that the ten bottles reserved for the final sampling time point as well as for ^13^CO_2_ measurements were 500 ml in volume for larger headspace to ensure oxic condition during the experiment. The microcosms were incubated at room temperature with shaking at 120 rpm. The 125-ml bottles were opened daily for 1-h air exchange. Sampled sludge was immediately frozen in liquid nitrogen and stored at -80 °C.

## Method S4 ^13^CO_2_ measurement

^13^CO_2_ production stemming from ^13^C-labelled bacterial biomass in microcosms was monitored via daily measurements with GC-MS (Trace1300-ISQ7000 GC-MS, ThermoFisher). For each measurement, 20 μl headspace gas sample was extracted with a gas-tight glass syringe and injected into the GC-MS. Gases were first separated by gas chromatography with a TG-BOND Q column at 40 °C (6 min) with helium as carrier gas (flow rate 1 ml min^-1^). Then molecular masses 44 Da (^12^CO_2_) and 45 Da (^13^CO_2_) were quantified with the ISQ7000 MS detector under selected ion mode (SIM). Data were analyzed using the software Chromeleon (version 7.2.10, Thermo Scientific). The relative abundance of ^13^CO_2_ in the headspace was calculated as the ratio of ^13^CO_2_ to total CO_2_ (^13^CO_2_ plus ^12^CO_2_).

## Method S5 RNA extraction and gradient centrifugation

Total RNA and genomic DNA was simultaneously extracted from 4 ml activated sludge of all the microcosms using RNeasy PowerSoil Total RNA Kit and RNeasy PowerSoil DNA Elution Kit (Qiagen) following the manufacturer’s instructions. The extracted RNA or DNA from the duplicate microcosms for each treatment at each sampling time point was pooled for the subsequent SIP and sequencing analyses. For the RNA extracts, residual DNA was removed using the TURBO DNA-free kit (Invitrogen) after pooling, and purified RNA was quantified with Qubit 4 using Qubit with RNA HS Assay Kit (Invitrogen).

SIP analysis was performed for samples collected after 16 hours, one day, two days, and four days of incubation. Gradients were prepared with 5 ml of 2 g ml^-1^ cesium trifluroacetate (CsTFA; GE Healthcare), 185 μl Hidi formamide (Applied Biosystems), and 1 ml of gradient buffer (0.1 M Tris-HCl pH 8.0, 0.1 M KCl, 1 mM EDTA) containing 1 μg of purified RNA. Gradients were well mixed and transferred into 5.1 ml seal tubes, sealed, and centrifuged on a P65VT2 rotor in a CP100NX ultracentrifuge (all Hitachi) at 125 000 g_av_ and 20 °C for ~64 h. Thirteen fractions (~500 μl) were collected from each gradient according to the procedures describe by Lueders [2], and density of the fractions was measured by weighing a 200 μl aliquot. Subsequently, RNA was retrieved from all the fractions by precipitation with isopropanol, washed with 70% ethanol, and resuspended in 25 μl TE.

## Method S6 Quantitative reverse transcription PCR

Quantitative distribution of the retrieved rRNA gene transcripts across the fractions of each gradient was determined with quantitative reverse transcription PCR (RT-qPCR) of 16S rRNA gene transcripts using the TaKaRa One Step PrimeScript RT-PCR Kit on a qTOWER3 thermal cycler (Analytik Jena). Each 40 μl RT-qPCR reaction contained 20 μl 2 ╳ One Step RT-PCR Buffer III, 0.8 μl TaKaRa Ex Taq HS, 0.8 μl PrimeScript RT Enzyme Mix II, 0.9 μM of each primer (Ba349F, 5’-AGGCAGCAGTDRGGAAT-3’; Ba806R, 5’-GGACTACYVGGGTATCTAAT-3’), and 0.3 μM Taqman probe (Ba516F, 5’-FAM-TGCCAGCAGCCGCGGTAATACRDAG-TAMRA-3’) [3]. rRNA standard was synthesized following the procedures described by Satinsky et al. [4] and Huang et al. [5] by linearization of a plasmid harboring the target region and vitro transcription using MEGAscript™ T7 Transcription Kit (Invitrogen), and used in RT-qPCR in a concentration series between 10^8^ and 10 copies μl^-1^.

## Method S7 Fraction sequencing and sequence analysis

The complementary DNA (cDNA) for the selected “heavy” (density 1.851-1.872 g ml^-1^) and “light” (density 1.805-1.819 g ml^-1^) fractions (Fig. S1) was synthesized using the TaKaRa PrimeScript II 1st Strand cDNA Synthesis Kit and the random primers following the manufacturer’s protocol. Bacterial 16S rRNA gene V3-V4 region and eukaryotic 18S rRNA gene V4 region were amplified from cDNA and the genomic DNA of all the microcosm sludge samples, using primer pairs 341F (5’- CCTAYGGGRBGCASCAG-3’) and 806R (5’-GGACTACNNGGGTATCTAAT-3’) with barcode, and 565F (5’-CCAGCASCYGCGGTAATTCC-3’) and 981R (5’-ACTTTCGTTCTTGATYRA-3’) with barcode, respectively. The pooled amplicon library was sequenced on the NovaSeq platform (Illumina) using a paired-end (2 ╳ 250 bp) sequencing strategy at the Guangdong Magigene Biotechnology Co., Ltd. (Guangzhou, China). The rRNA gene amplicon sequence data were processed using QIIME 2 pipeline [6] and DADA2 algorithm [7], generating amplicon sequence variant (ASV) tables based on 100% sequence identity for both 16S rRNA and 18S rRNA genes. Taxonomy was assigned to ASVs using the SILVA SSU database (version 138) [8]. The ASVs affiliated to chloroplasts or mitochondria were removed, and the ASV tables were rarefied to an even sequence depth of 51 302 and 40 508 for the 16S and 18S rRNA gene dataset, composing 16 671 bacterial and 5 258 eukaryotic ASVs, respectively.

## Method S8 WWTP sludge DNA extraction, sequencing, and data analyses

Genomic DNA was extracted from 2-ml aliquots of the aerobic and anaerobic sludge samples collected from the local WWTP after centrifugation at 20 817 g and 4 °C for 5 min using FastDNA Spin Kit for Soil (MP Medicals). Bacterial 16S rRNA gene V3-V4 regions (341F, 5’-CCTAYGGGRBGCASCAG-3’; 806R, 5’-GGACTACNNGGGTATCTAAT-3’) were amplified and sequenced on the NovaSeq platform producing 250 bp paired-end reads at the Guangdong Magigene Biotechnology Co., Ltd. (Guangzhou, China). Sequencing data were processed following the same procedures as for the SIP gradient fractions and microcosm DNA (Method S6), with the final ASV table containing 7 841 ASVs from an even sequence depth of 22 646.

Also, full-length bacterial 16S rRNA genes were amplified from the aerobic and anaerobic sludge DNA, using primer pairs 341F (5’-CCTAYGGGRBGCASCAG-3’) and 806R (5’-GGACTACNNGGGTATCTAAT-3’) with barcode. Amplicons were mixed and purified, followed by sequencing library preparation according to the 16S Amplification SMRTbell Library Preparation workflow and sequencing on Sequel II platform (PacBio). Sequencing data was first processed with SMRT link (version 6.0) software, including data splitting and sequence error correction. Clean sequences were clustered into operational taxonomic units (OTUs) based on 99% sequence identity using the USEARCH software (v10), generating 15 274 OTUs. Taxonomy was assigned to OTUs using QIIME 2 [6] with the SILVA SSU database (version 138) [8].

The difference in relative sequence abundance of *Myxococcota* and *Bdellovibrionota* between aerobic and anaerobic sludges, including the putative predatory bacteria identified in the SIP-microcosm experiment, was evaluated by applying paired samples Wilcoxon test using PAST (version 4.03).

## Method S9 Global WWTP full-length bacterial 16S rRNA gene sequences and phylogenetic analyses

To characterize the diversity of *Myxococcota* and *Bdellovibrionota* species in WWTPs, representative full-length 16S rRNA gene sequences of *Myxococcota* (507) and *Bdellovibrionota* (105) OTUs obtained in this study were extracted and combined with sequences acquired from the MiDAS database, a global catalogue of WWTP bacterial 16S rRNA gene sequences [9]. There exist in MiDAS 4.0 a total of 2 559 ASVs assigned to *Myxococcota* and 1 979 ASVs to *Bdellovibrionota*. The MiDAS ASV sequences were first clustered into OTUs using USEARCH (99% sequence identity cutoff, resulting in 1 010 and 1 233 OTUs for *Myxococcota* and *Bdellovibrionota*, respectively), before all the representative OTU sequences were aligned using the software MAFFT (v7.471) with default settings, and phylogenetic trees were constructed using the maximum likelihood approach with the default parameters of FastTree (version 2.1.10 Double precision). For *Myxococcota*, we established a collection of 129 high-quality 16S rRNA gene sequences of cultured *Myxococcota* with experimental evidence of predatory capability reported in literature (Table S2), on the basis of a comprehensive dataset of myxobacterial 16S rRNA gene sequences constructed by Liu et al [10] from the NCBI database, SILVA database [11], RDP database [12], and IMG database [13]. Based on results of blastn (version 2.10.1+) against the sequences of cultured isolates with observational evidence of predatory capability, the myxobacterial 16S rRNA gene sequences in the phylogenetic tree were annotated as potential predators at genus (sequence identity > 94.5%) level. Further phylogenetic analysis was applied to selected myxobacterial genera (*Haliangium*, the mle1-27 clade, *Pajaroellobacter*, *Nannocystis*, and *Polyangium*) to explore their cross-habitat diversity by combining the full-length the 16S rRNA gene sequences of WWTP01 with sequences (> 1 200 bp) from the SILVA database [8] with known isolation source, following the same alignment and phylogenetic tree construction procedures as described above. All the phylogenetic trees were visualized and annotated in Interactive Tree Of Life (iTOL) [14].

## Method S10 Global activated sludge microbiome dataset processing and statistical analyses

We employed an activated sludge microbiome dataset of global WWTPs published by the Global Water Microbiome Consortium [15] to uncover the global distribution of *Myxococcota* and *Bdellovibrionota* in activated sludge. The Global Water Microbiome Consortium systematically sampled and investigated the global activated sludge prokaryotic communities of 1 186 sludge samples from 269 WWTPs in 23 countries on 6 continents, covering diverse climate zones, spatial scales, and activated sludge process types [15], based on amplicon sequencing of 16S rRNA gene V4 region. By defining OTUs at 97% sequence identity threshold, a total of 96 148 OTUs were obtained in this study. We applied taxonomy assignment to representative sequences using the SILVA SSU database (version 138) [8] in QIIME 2 [6]. Specifically, abundance data of *Myxococcota* and *Bdellovibrionota* as well as the abundant genera were extracted and investigated for the correlation to sludge performance, abundance of functional bacteria, and sludge process parameters, by computing Spearman’s rank correlation with *p*-values corrected for multiple testing with the Benjamini-Hochberg method in PAST (version 4.03). Activated sludge function was represented by the removal rates (g per g biomass per day) of BOD (biochemical oxygen demand), COD (chemical oxygen demand), ammonia nitrogen (NH_4_-N), total nitrogen (TN), and total phosphorus (TP), as described by Wu et al., and calculated as follows [15]:

$Removal rate=\frac{\left( C_{\mathrm{Influent}}-C_{\mathrm{Effluent}} \right) \times flow rate}{MLSS \times aerobic tank volume}$ (1)

where C_Influent_ and C_Effluent_ are the pollutant concentrations in influent and effluent samples, and MLSS is mixed liquor suspended solids.

# Supplementary Figures


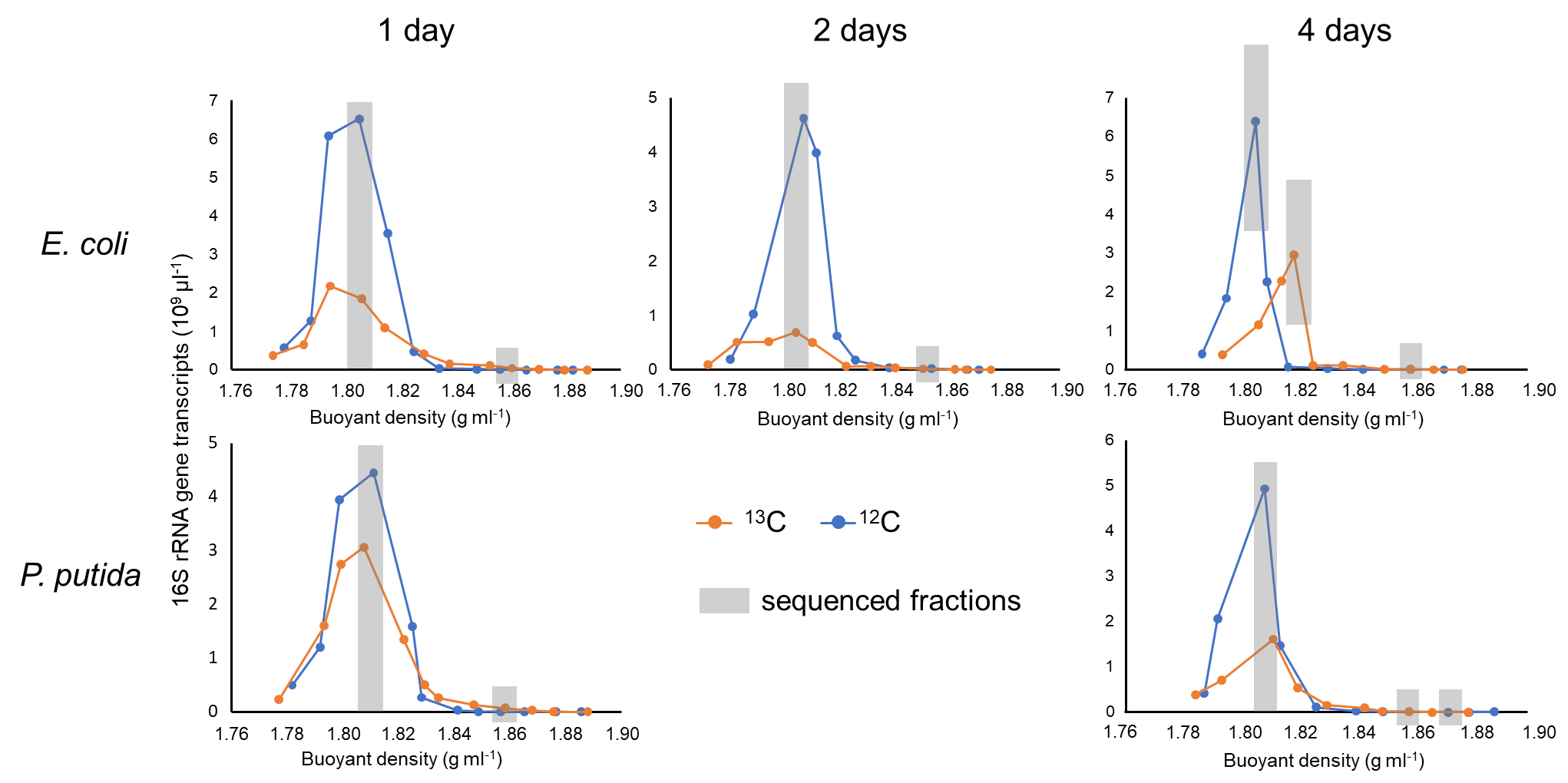


**Fig. S1** Copies of 16S rRNA gene transcripts across the entire buoyant density gradient of the RNA extracted from activated sludge microcosms amended with *E. coli* and *P. putida* cells after one, two, and four days of incubation. The 16S rRNA gene transcripts were quantified using real-time reverse transcription PCR. The rRNA fractions marked with grey background were subjected to the subsequent amplicon sequencing analysis.


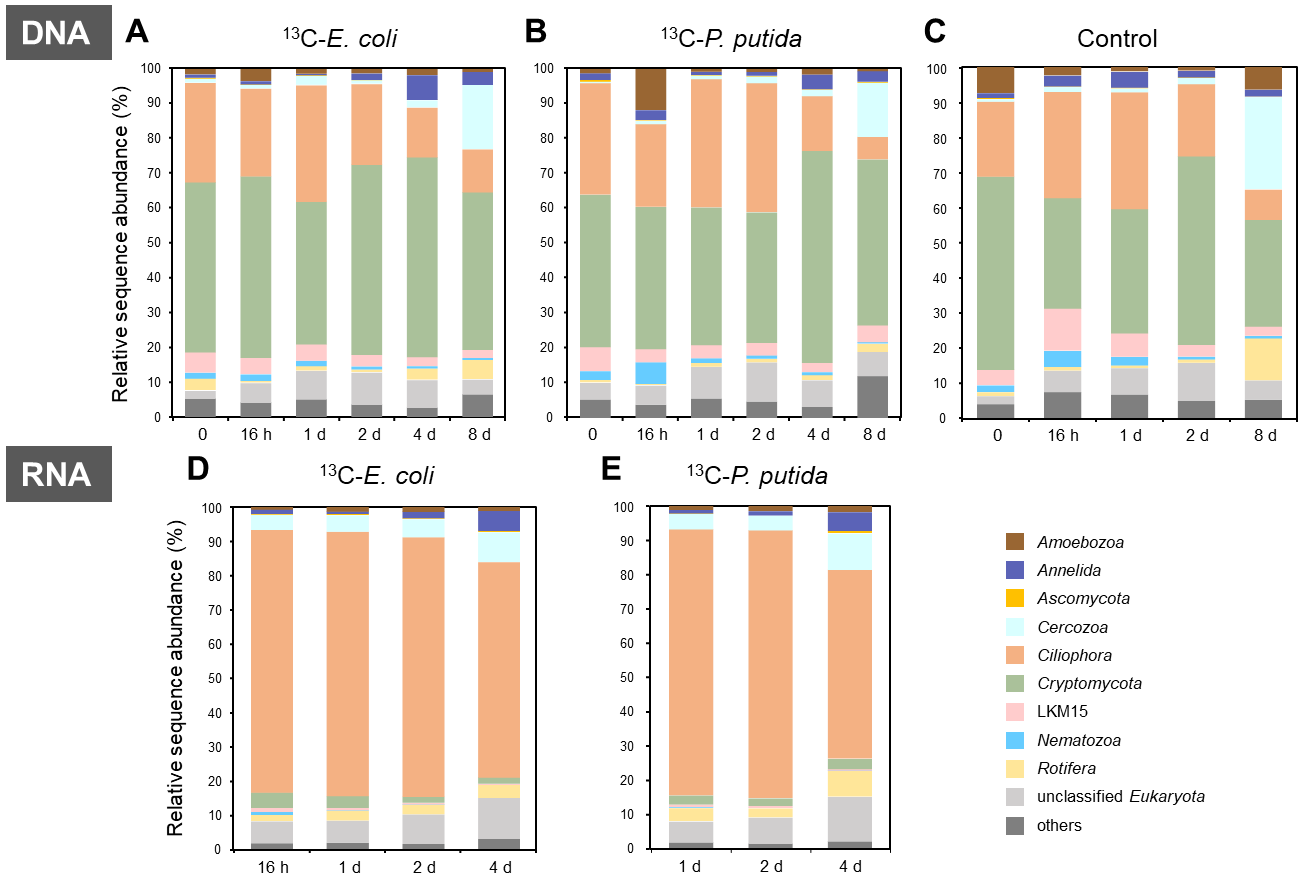


**Fig. S2** The dynamics of the micro-eukaryotic communities during the microcosm experiment. Structure of the active micro-eukaryotic communities was inferred based on amplicon sequencing of the light rRNA fractions from the microcosms amended with ^13^C-*E. coli* (A) and ^13^C-*P. putida* (B) cells. The overall micro-eukaryotic communities were obtained by 18S rRNA gene amplicon sequencing of the total DNA from the activated sludge microcosms amended with ^13^C-*E. coli* (C) and ^13^C-*P. putida* (D) cells, and the control group (E) without amendment. Relative sequence abundance of the ten most abundant micro-eukaryotic phyla was shown.


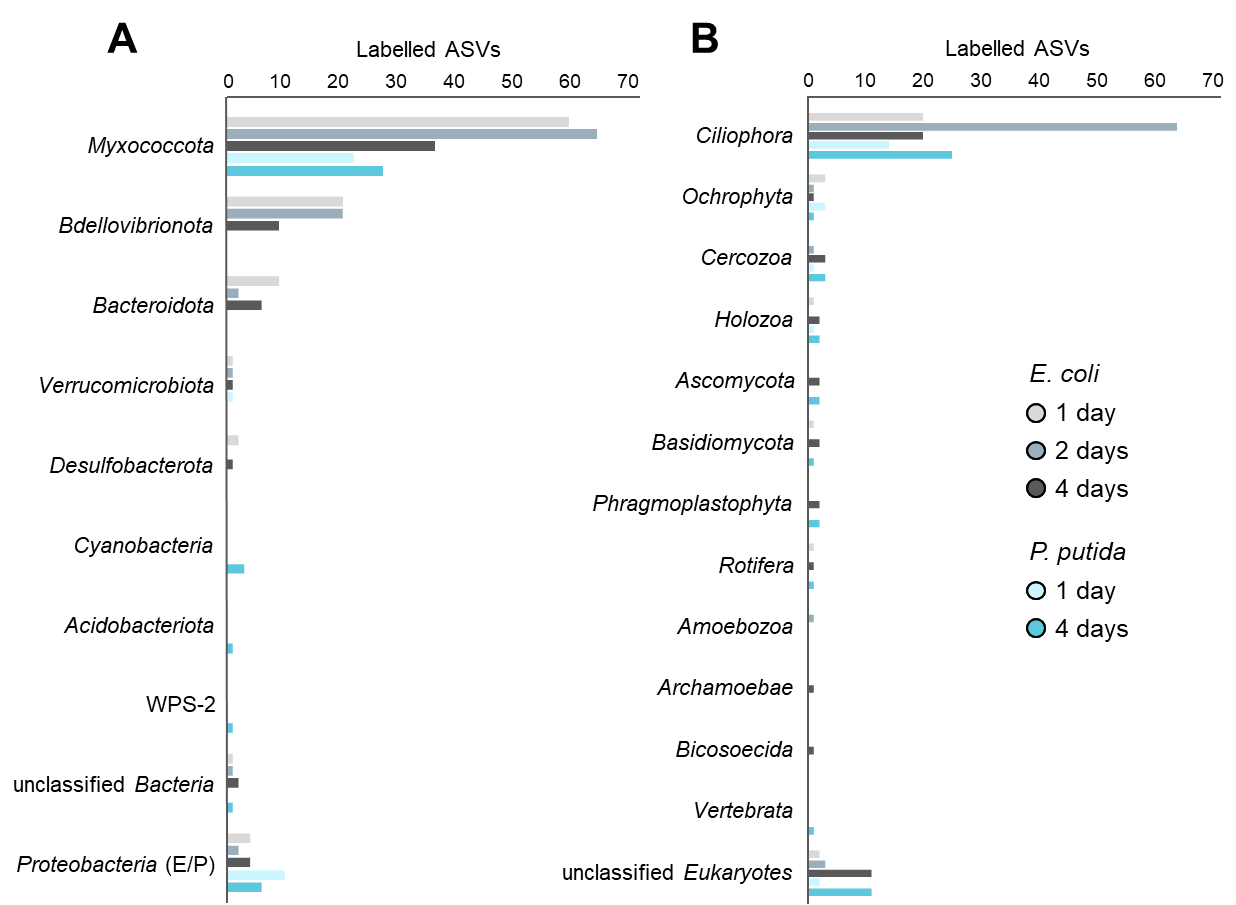


**Fig. S3** Taxonomic diversity of the ^13^C-incorporators identified by SIP. Labelled prokaryotic (A) and micro-eukaryotic (B) ASVs were determined with an EF threshold > 0.1, and only ASVs with relative sequence abundance > 0.1% in the heavy rRNA fractions of at least one of the ^13^C-*E. coli* and ^13^C-*P. putida* microcosms at a sampling point were counted. The labelled proteobacterial ASVs were exclusively affiliated with *Escherichia-Shigella* or *Pseudomonas* (E/P).

**
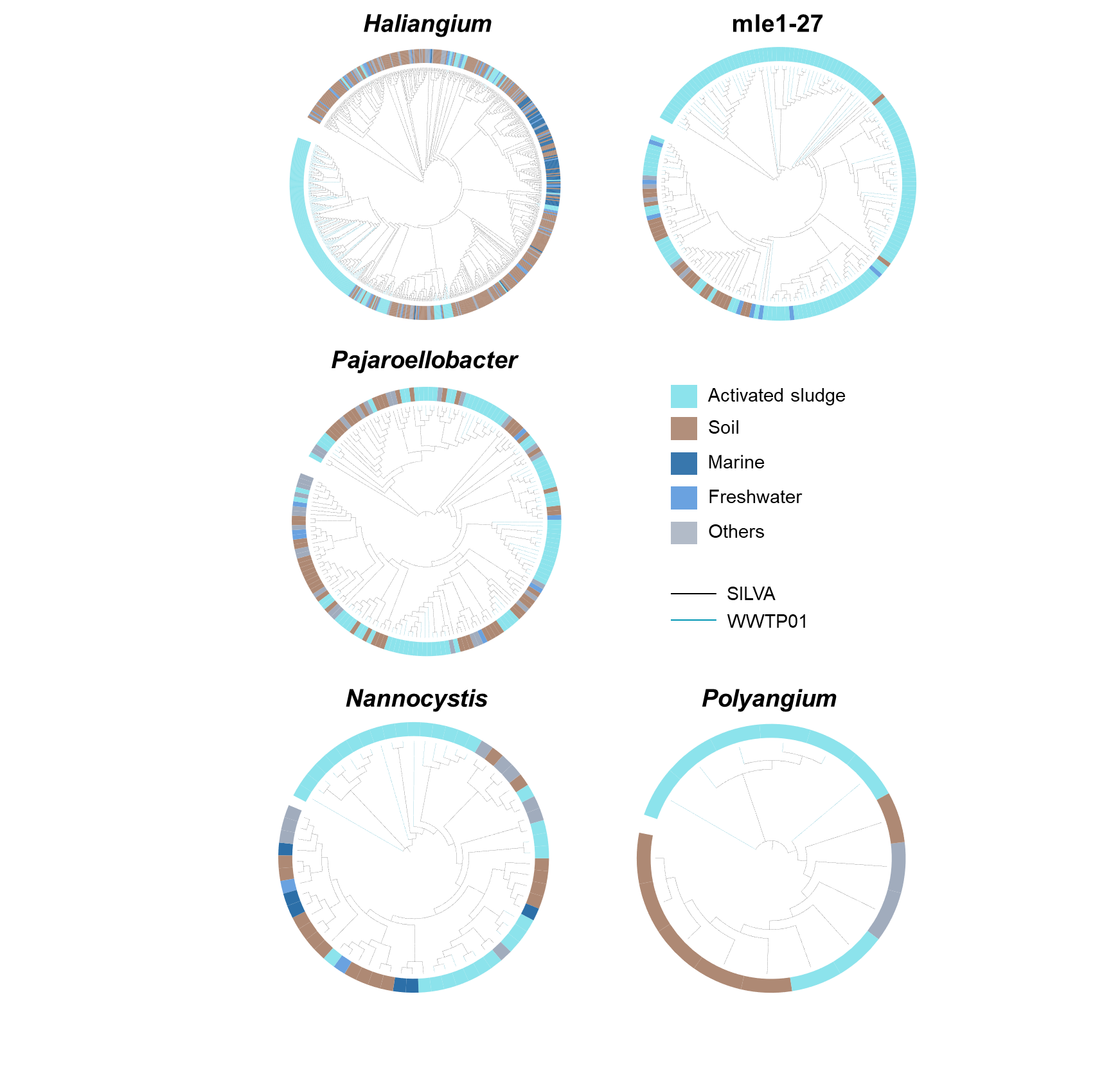
**

**Fig. S4** Phylogenetic trees inferred for selected myxobacterial genera from near full-length 16S rRNA gene sequences across different habitats. The trees were constructed with representative OTU sequences from a local WWTP (174, 134, 69, 27, and 6 sequences, for *Haliangium*, the mle1-27 clade, *Pajaroellobacter*, *Nannocystis*, and *Polyangium*, respectively) and sequences obtained from the SILVA database [8] with known isolation source (507, 49, 102, 38, and 10 sequences, respectively).

**
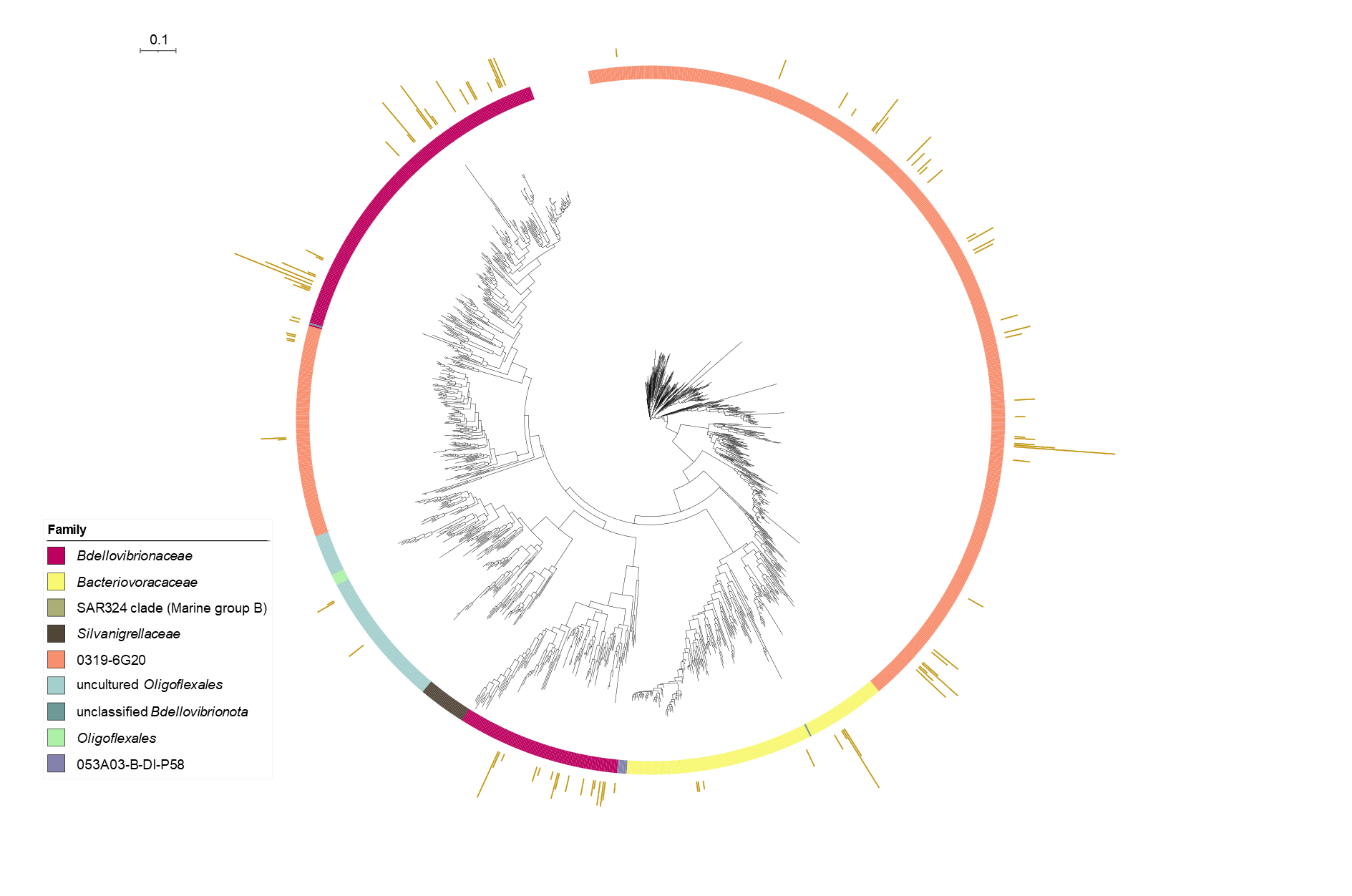
Fig. S5** Phylogenetic tree inferred from full-length 16S rRNA gene sequences within the phylum *Bdellovibrionota* originated from global WWTPs. The tree was constructed with 105 representative OTU sequences from a local WWTP (WWTP01) and 1 233 reference sequences from the MiDAS 4 database [9]. The external bars showed average relative abundance of the OTUs at the WWTP01 across eight aerobic activated sludge samples collected over two years (maximum 0.014%). The scale bar corresponds to 0.1 substitutions per nucleotide position.


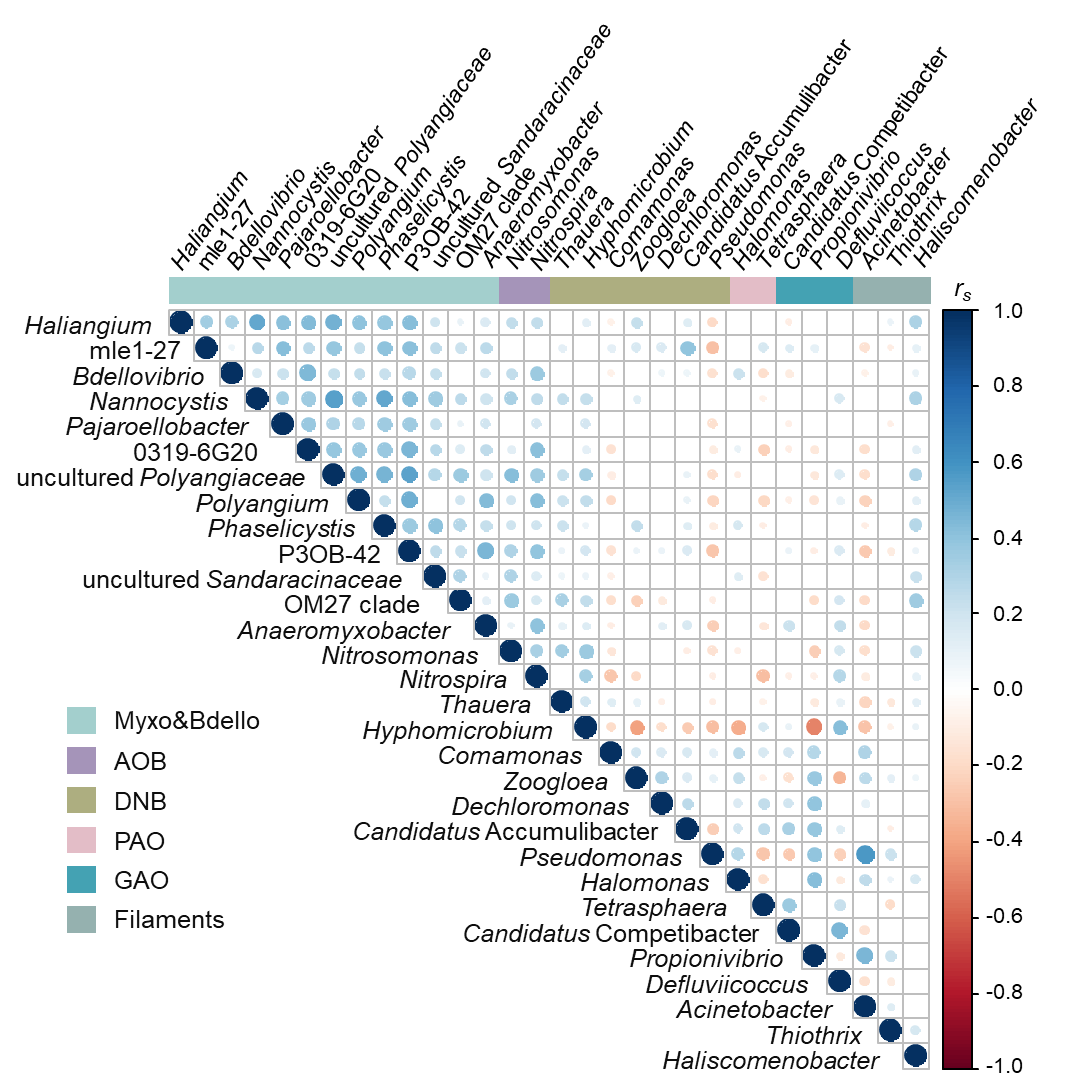


**Fig. S6** The correlation between members of *Myxococcota* and *Bdellovibrionota* and sludge performance-relevant bacteria in global activated sludge, evaluated using data acquired from the Global Water Microbiome Consortium [15] of 1 186 activated sludge samples from 269 WWTPs across 23 countries. Color and size of the circles indicate Spearman’s rank correlation coefficients, and circles were displayed only for significant correlation (*p* < 0.05, n = 1 186). Correlation among only selected *Myxococcota* and *Bdellovibrionota* genus-level taxa, as well as sludge performance-relevant bacteria with average relative sequence abundance > 0.1% across all the 1 186 samples were analyzed and displayed. AOB, ammonia-oxidizing bacteria; DNB, denitrifying bacteria; PAO, polyphosphate-accumulating organism; GAO, glycogen-accumulating organism.


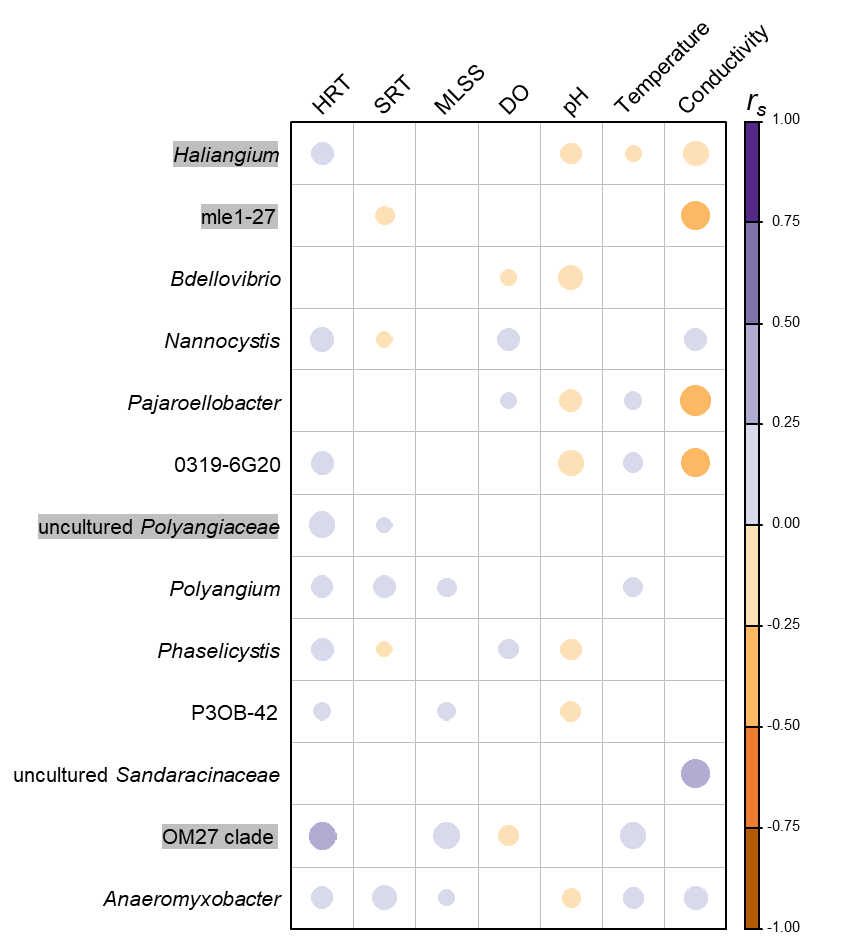


**Fig. S7** The impact of sludge process parameters on predatory bacteria in activated sludge, evaluated using data acquired from the Global Water Microbiome Consortium [15] of 1 186 activated sludge samples worldwide. Color and size of the circles indicate Spearman’s rank correlation coefficients, and circles were displayed only for significant correlation (*p* < 0.05, n = 634, 844, 765, 927, 869, 787, and 248, for HRT, SRT, MLSS, DO, pH, temperature, and conductivity, respectively, corrected with the Benjamini-Hochberg method for multiple testing). Only the genus-level taxa with average relative sequence abundance > 0.1% across all the 1 186 samples are shown, with the putative predators identified by SIP in microcosm experiment marked with grey background. HRT, aeration tank hydraulic retention time; SRT, solid retention time; MLSS, mixed liquor suspended solids; DO, dissolved oxygen.

# Supplementary Tables

**Table S1** Sampling dates from March 2019 to April 2021. Sludge samples were collected from the aeration and anaerobic tanks at the same sampling points.

| **No.** | **Date** |
| --- | --- |
| 1 | 3-March-2019 |
| 2 | 23-July-2019 |
| 3 | 19-August-2019 |
| 4 | 5-November-2019 |
| 5 | 28-May-2020 |
| 6 | 21-October-2020 |
| 7 | 28-January-2021 |
| 8 | 20-April-2021 |

**Table S2** Source information of the collected sequences of *Myxococcota* isolates with reported experimental evidence of predatory capability.

| Accession No. | Isolation source | Pubmed/DOI | Sequence Type |
| --- | --- | --- | --- |
| AB016469 | Wet sand | 9868776 | 16S rRNA gene |
| AF336801 | Seawater | 11997170 | 16S rRNA gene |
| AB062751 | Coastal sand | 12469307 | 16S rRNA gene |
| AB083432 | Seagrass | 12656172 | 16S rRNA gene |
| ABCS01000137 | Seagrass | 12656172 | genome |
| AB097591 | Wet sand | 12866845 | 16S rRNA gene |
| AB097590 | Coastal mud | 12866845 | 16S rRNA gene |
| AY996786 | Garden soil | 16156733 | 16S rRNA gene |
| AY996785 | Garden soil | 16156733 | 16S rRNA gene |
| AY996784 | Garden soil | 16156733 | 16S rRNA gene |
| AY996783 | Garden soil | 16156733 | 16S rRNA gene |
| AY996782 | Garden soil | 16156733 | 16S rRNA gene |
| AB252740 | Coastal soil | 17025014 | 16S rRNA gene |
| DQ105523 | Coastal sediment | 17186141 | 16S rRNA gene |
| DQ105524 | Coastal sediment | 17186141 | 16S rRNA gene |
| DQ105522 | Coastal sediment | 17186141 | 16S rRNA gene |
| DQ105521 | Coastal sediment | 17186141 | 16S rRNA gene |
| AY072740 | Coastal soil | 17186141 | 16S rRNA gene |
| AY032879 | Sediment | 17186141 | 16S rRNA gene |
| AF466191 | Coastal soil | 17186141 | 16S rRNA gene |
| DQ105525 | Seawater | 17186141 | 16S rRNA gene |
| DQ105526 | Seawater | 17186141 | 16S rRNA gene |
| AB016470 | Dry seaweed | 21304682 | 16S rRNA gene |
| GU323923 | Marine sediment | 22189493 | 16S rRNA gene |
| GU323922 | Marine sediment | 22189493 | 16S rRNA gene |
| AB600195 | Mud | 22821734 | 16S rRNA gene |
| KF767690 | Soil | 24591423 | 16S rRNA gene |
| KT070190 | Soil | 26214047 | 16S rRNA gene |
| KP718975 | Sand | 26669488 | 16S rRNA gene |
| KP718976 | Compost | 26669488 | 16S rRNA gene |
| KP718977 | Compost | 26669488 | 16S rRNA gene |
| KP718979 | Compost | 26669488 | 16S rRNA gene |
| KP718974 | Sand | 26669488 | 16S rRNA gene |
| KP718978 | Compost | 26669488 | 16S rRNA gene |
| CP006003 | Soil | 26734118 | genome |
| KT591707 | Soil | 27046779 | 16S rRNA gene |
| KX430041 | Soil | 28141508 | 16S rRNA gene |
| KX430042 | Soil | 28141508 | 16S rRNA gene |
| KY381122 | Soil | 29458458 | 16S rRNA gene |
| PVNK01000078 | Sediment | 29700155 | 16S rRNA gene |
| PVNL01000027 | Sediment | 29700155 | 16S rRNA gene |
| MG821450 | Soil | 30234476 | 16S rRNA gene |
| MG824978 | Unknown | 30234476 | 16S rRNA gene |
| MG821449 | Soil | 30234476 | 16S rRNA gene |
| MG821410 | Soil | 30234476 | 16S rRNA gene |
| MG821430 | Soil | 30234476 | 16S rRNA gene |
| MG821439 | Soil | 30234476 | 16S rRNA gene |
| MG821411 | Soil | 30234476 | 16S rRNA gene |
| MG821426 | Soil | 30234476 | 16S rRNA gene |
| MG821457 | Soil | 30234476 | 16S rRNA gene |
| MG821401 | Unknown | 30234476 | 16S rRNA gene |
| MG824983 | Soil | 30234476 | 16S rRNA gene |
| MG821416 | Soil | 30234476 | 16S rRNA gene |
| MG821462 | Soil | 30234476 | 16S rRNA gene |
| MG821456 | Soil | 30234476 | 16S rRNA gene |
| MG821451 | Sand | 30234476 | 16S rRNA gene |
| MG821443 | Soil | 30234476 | 16S rRNA gene |
| MG824984 | Soil | 30234476 | 16S rRNA gene |
| MG821452 | Soil | 30234476 | 16S rRNA gene |
| MG821445 | Soil | 30234476 | 16S rRNA gene |
| MG821448 | Soil | 30234476 | 16S rRNA gene |
| MG821402 | Soil | 30234476 | 16S rRNA gene |
| MG821437 | Faeces from herbivores | 30234476 | 16S rRNA gene |
| MG824981 | Soil | 30234476 | 16S rRNA gene |
| MG821464 | Soil | 30234476 | 16S rRNA gene |
| MG821406 | Soil | 30234476 | 16S rRNA gene |
| MG821453 | Unknown | 30234476 | 16S rRNA gene |
| MG821424 | Soil | 30234476 | 16S rRNA gene |
| MG821461 | Soil | 30234476 | 16S rRNA gene |
| MG821414 | Soil | 30234476 | 16S rRNA gene |
| MG821417 | Soil | 30234476 | 16S rRNA gene |
| MG821419 | Soil | 30234476 | 16S rRNA gene |
| MG821427 | Soil | 30234476 | 16S rRNA gene |
| MG821434 | Soil | 30234476 | 16S rRNA gene |
| MG821454 | Sand | 30234476 | 16S rRNA gene |
| MG824980 | Soil | 30234476 | 16S rRNA gene |
| MG821404 | Soil | 30234476 | 16S rRNA gene |
| MG821423 | Soil | 30234476 | 16S rRNA gene |
| MG824979 | Mud from rice field | 30234476 | 16S rRNA gene |
| MG821403 | Unknown | 30234476 | 16S rRNA gene |
| MG821415 | Soil | 30234476 | 16S rRNA gene |
| MG821407 | Soil | 30234476 | 16S rRNA gene |
| MG821431 | Soil | 30234476 | 16S rRNA gene |
| MG821433 | Soil | 30234476 | 16S rRNA gene |
| MG821455 | Soil | 30234476 | 16S rRNA gene |
| MG821436 | Mud from rice field | 30234476 | 16S rRNA gene |
| MG821444 | Soil | 30234476 | 16S rRNA gene |
| MG821458 | Soil | 30234476 | 16S rRNA gene |
| MG821441 | Soil | 30234476 | 16S rRNA gene |
| MG821442 | Soil | 30234476 | 16S rRNA gene |
| MG821405 | Soil | 30234476 | 16S rRNA gene |
| MG821429 | Soil | 30234476 | 16S rRNA gene |
| MG821459 | Soil | 30234476 | 16S rRNA gene |
| MG821460 | Soil | 30234476 | 16S rRNA gene |
| MG821438 | Soil | 30234476 | 16S rRNA gene |
| MG821408 | Soil | 30234476 | 16S rRNA gene |
| MG821465 | Mud from rice field | 30234476 | 16S rRNA gene |
| MG821425 | Soil | 30234476 | 16S rRNA gene |
| MG821446 | Soil | 30234476 | 16S rRNA gene |
| MG821435 | Soil | 30234476 | 16S rRNA gene |
| MG821420 | Soil | 30234476 | 16S rRNA gene |
| MG824982 | Soil | 30234476 | 16S rRNA gene |
| MG821413 | Soil | 30234476 | 16S rRNA gene |
| MG821428 | Soil | 30234476 | 16S rRNA gene |
| MG821463 | Soil | 30234476 | 16S rRNA gene |
| MG821409 | Sand | 30234476 | 16S rRNA gene |
| MG821422 | Bark of olive tree | 30234476 | 16S rRNA gene |
| MG821418 | Soil | 30234476 | 16S rRNA gene |
| MG821440 | Soil | 30234476 | 16S rRNA gene |
| MG821412 | Soil | 30234476 | 16S rRNA gene |
| MG821421 | Soil | 30234476 | 16S rRNA gene |
| MG821432 | Soil | 30234476 | 16S rRNA gene |
| MG821447 | Soil | 30234476 | 16S rRNA gene |
| KX810170 | Soil | 10.1002/mbo3.464 | 16S rRNA gene |
| KX810185 | Rabbit feces | 10.1002/mbo3.464 | 16S rRNA gene |
| KX810190 | Soil | 10.1002/mbo3.464 | 16S rRNA gene |
| KX810188 | Boar feces | 10.1002/mbo3.464 | 16S rRNA gene |
| KX810169 | Deer feces | 10.1002/mbo3.464 | 16S rRNA gene |
| KX256198 | Soil | 10.1007/s12223-017-0502-2 | 16S rRNA gene |
| KX256197 | Soil | 10.1007/s12223-017-0502-2 | 16S rRNA gene |
| AB246767 | Hot spring biomat | 10.1264/jsme2.21.189 | 16S rRNA gene |
| AB246768 | Hot spring biomat | 10.1264/jsme2.21.189 | 16S rRNA gene |
| AB246770 | Hot spring biomat | 10.1264/jsme2.21.189 | 16S rRNA gene |
| AB246769 | Coastal hot spring sand | 10.1264/jsme2.21.189 | 16S rRNA gene |
| AB246772 | Hot spring biomat | 10.1264/jsme2.21.189 | 16S rRNA gene |
| AB246771 | Hot spring biomat | 10.1264/jsme2.21.189 | 16S rRNA gene |
| HM769729 | Sediment | 10.3390/md8092466 | 16S rRNA gene |
| HM769728 | Sediment | 10.3390/md8092466 | 16S rRNA gene |
| HM769727 | Sediment | 10.3390/md8092466 | 16S rRNA gene |

# References

1. Zhang Z, Zhang G, Ju F. Using culture-enriched phenotypic metagenomics for targeted high-throughput monitoring of the clinically important fraction of the beta-lactam resistome. Environ Sci Technol. 2022; 56(16):11429–11439.

2. Lueders T. DNA- and RNA-based stable isotope probing of hydrocarbon degraders. In: McGenity TJ, Timmis KN, Nogales B (eds). Hydrocarbon and Lipid Microbiology Protocols: Genetic, Genomic and System Analyses of Communities. Springer Protocols Handbooks. Springer, Berlin, Heidelberg, 2015; 181–197.

3. Takai K, Horikoshi K. Rapid detection and quantification of members of the archaeal community by quantitative PCR using fluorogenic probes. Appl Environ Microbiol. 2000; 66(11):5066-5072.

4. Satinsky BM, Gifford SM, Crump BC, Moran MA. Chapter Twelve - Use of Internal Standards for Quantitative Metatranscriptome and Metagenome Analysis. In: DeLong EF, editor. Methods in Enzymology. 531. Academic Press, 2013; 237-250.

5. Huang XY, Zhang L, Yuan L, Ju F. Quantitative Metagenomics and Metatranscriptomics Methods of Microbiome. Bio-protocol. 2021; e2003693.

6. Bolyen E, Rideout JR, Dillon MR, Bokulich NA, Abnet CC, Al-Ghalith GA, *et al*. Reproducible, interactive, scalable and extensible microbiome data science using QIIME 2. Nat Biotechnol. 2019; 37:852–857.

7. Callahan BJ, McMurdie PJ, Rosen MJ, Han AW, Johnson AJA, Holmes SP. DADA2: High-resolution sample inference from Illumina amplicon data. Nature Methods. 2016; 13(7):581–583.

8. Quast C, Pruesse E, Yilmaz P, Gerken J, Schweer T, Yarza P, *et al*. The SILVA ribosomal RNA gene database project: improved data processing and web-based tools. Nucleic Acids Res. 2013; 41(D1):D590–D596.

9. Dueholm MKD, Nierychlo M, Andersen KS, Rudkjøbing V, Knutsson S, Arriaga S, *et al*. MiDAS 4: A global catalogue of full-length 16S rRNA gene sequences and taxonomy for studies of bacterial communities in wastewater treatment plants. Nat Commun. 2022; 13:1908.

10. Liu Y, Yao Q, Zhu H. Meta-16S rRNA gene phylogenetic reconstruction reveals the astonishing diversity of cosmopolitan myxobacteria. Microorganisms. 2019; 7(11):551.

11. Pruesse E, Quast C, Knittel K, Fuchs BM, Ludwig W, Peplies J, et al. SILVA: a comprehensive online resource for quality checked and aligned ribosomal RNA sequence data compatible with ARB. Nucleic Acids Res. 2007; 35(21):7188-7196.

12. Cole JR, Wang Q, Fish JA, Chai B, McGarrell DM, Sun Y, et al. Ribosomal Database Project: data and tools for high throughput rRNA analysis. Nucleic Acids Res. 2014; 42(D1):D633-D642.

13. Markowitz VM, Chen IMA, Palaniappan K, Chu K, Szeto E, Grechkin Y, et al. IMG: the integrated microbial genomes database and comparative analysis system. Nucleic Acids Res. 2011; 40(D1):D115-D122.

14. Letunic I, Bork P. Interactive tree of life (iTOL) v3: an online tool for the display and annotation of phylogenetic and other trees. Nucleic Acids Res. 2016; 44(W1):W242-W245.

15. Wu L, Ning D, Zhang B, Li Y, Zhang P, Shan X, et al. Global diversity and biogeography of bacterial communities in wastewater treatment plants. Nat Microbiol. 2019; 4:1183-1195.
